# Pyruvate Homeostasis as a Determinant of Parasite Growth and Metabolic Plasticity in Toxoplasma gondii

**DOI:** 10.1128/mBio.00898-19

**Published:** 2019-06-11

**Authors:** Ningbo Xia, Shu Ye, Xiaohan Liang, Pu Chen, Yanqin Zhou, Rui Fang, Junlong Zhao, Nishith Gupta, Shuzhen Yang, Jing Yuan, Bang Shen

**Affiliations:** a State Key Laboratory of Agricultural Microbiology, College of Veterinary Medicine, Huazhong Agricultural University, Wuhan, Hubei Province, People’s Republic of China; b Hubei Cooperative Innovation Center for Sustainable Pig Production, Wuhan, Hubei Province, People’s Republic of China; c Key Laboratory of Preventive Medicine in Hubei Province, Wuhan, Hubei Province, People’s Republic of China; d Institute of Biology, Faculty of Life Sciences, Humboldt University, Berlin, Germany; e State Key Laboratory of Cellular Stress Biology, Innovation Center for Cell Signal Network, School of Life Sciences, Xiamen University, Xiamen, Fujian Province, People’s Republic of China; Drexel University College of Medicine; Washington University School of Medicine

**Keywords:** *Toxoplasma*, apicoplast, lactate, metabolic flexibility, pyruvate kinase

## Abstract

Toxoplasma gondii is a widespread intracellular pathogen infecting humans and a variety of animals. Previous studies have shown that *Toxoplasma* uses glucose and glutamine as the main carbon sources to support asexual reproduction, but neither nutrient is essential. Such metabolic flexibility may allow it to survive within diverse host cell types. Here, by focusing on the glycolytic enzyme pyruvate kinase (PYK) that converts phosphoenolpyruvate (PEP) into pyruvate, we found that *Toxoplasma* can also utilize lactate and alanine. We show that catabolism of all indicated carbon sources converges at pyruvate, and maintaining a constant pyruvate supply is critical to parasite growth. *Toxoplasma* expresses two PYKs: PYK1 in the cytosol and PYK2 in the apicoplast (a chloroplast relict). Genetic deletion of *PYK2* did not noticeably affect parasite growth and virulence, which contrasts with the current model of carbon metabolism in the apicoplast. On the other hand, *PYK1* was refractory to disruption. Conditional depletion of PYK1 resulted in global alteration of carbon metabolism, amylopectin accumulation, and reduced cellular ATP, leading to severe growth impairment. Notably, the attenuated growth of the PYK1-depleted mutant was partially rescued by lactate or alanine supplementation, and rescue by lactate required lactate dehydrogenase activity to convert it to pyruvate. Moreover, depletion of PYK1 in conjunction with PYK2 ablation led to accentuated loss of apicoplasts and complete growth arrest. Together, our results underline a critical role of pyruvate homeostasis in determining the metabolic flexibility and apicoplast maintenance, and they significantly extend our current understanding of carbon metabolism in T. gondii.

## Abstract (precis)

Toxoplasma gondii infects almost all warm-blooded animals, and metabolic flexibility is deemed critical for its successful parasitism in diverse hosts. Glucose and glutamine are the major carbon sources to support parasite growth. In this study, we found that *Toxoplasma* is also competent in utilizing lactate and alanine and, thus, exhibits exceptional metabolic versatility. Notably, all these nutrients need to be converted to pyruvate to fuel the lytic cycle, and achieving a continued pyruvate supply is vital to parasite survival and metabolic flexibility. Although pyruvate can be generated by two distinct pyruvate kinases, located in cytosol and apicoplast, respectively, the cytosolic enzyme is the main source of subcellular pyruvate, and cooperative usage of pyruvate among multiple organelles is critical for parasite growth and virulence. These findings expand our current understanding of carbon metabolism in Toxoplasma gondii and related parasites while providing a basis for designing novel antiparasitic interventions.

## INTRODUCTION

Toxoplasma gondii is an obligate intracellular parasite belonging to the phylum *Apicomplexa*, which comprises over 6,000 pathogen species of medical and veterinary importance, such as those of *Plasmodium*, *Cryptosporidium, Neospora*, and *Eimeria* (1). *Toxoplasma* can infect and reproduce in a range of warm-blooded animals (2). It is therefore considered one of the most successful pathogens. It causes severe toxoplasmosis in neonates and immune-compromised individuals, and about one-third of the global human population is seropositive to T. gondii (2, 3).

One unique aspect of *Toxoplasma* is its ability to reversibly switch between acute and chronic stages, known as tachyzoites and bradyzoites, respectively (4), which is a significant contributor to the pathogenesis and transmission of this parasite (5–7). While the chronic infection involves latent, often lifelong persistence of bradyzoites enclosed within tissue cysts, acute infection is caused by rapid intracellular proliferation and subsequent tissue necrosis by tachyzoites (5–7). Its reproduction in diverse host cell types demands an adjustable metabolic capacity to meet constant supply of energy and biomass (proteins, lipids, nucleotides) irrespective of the nutritional environments (8–11). Tachyzoites of T. gondii can utilize glucose and glutamine as the major carbon and energy sources (10, 12, 13). The parasite encodes all major pathways of central carbon metabolism, including glycolysis and gluconeogenesis in the cytosol, as well as the tricarboxylic acid (TCA) cycle and oxidative phosphorylation in the mitochondrion (10, 14–16). Previous work has demonstrated that either glucose or glutamine is sufficient to maintain parasite survival and virulence (10, 12, 13). It is suggested that having such a nutritional flexibility leverages parasite growth in different milieus as well as in different host cell types (10).

Although glucose is a major nutrient for T. gondii, its glucose transporter (GT1) and the first glycolytic enzyme, hexokinase (HK), are dispensable for tachyzoite reproduction (12, 17). The parasite can no longer catabolize host-derived glucose through glycolysis in the absence of GT1 or HK, and it becomes strictly dependent on glutamine to sustain its bioenergetic requirements (12, 17). Glutamine enters the TCA cycle to produce energy via oxidative phosphorylation as well as to replenish glycolytic intermediates for macromolecular synthesis via gluconeogenesis (10, 12, 13). The otherwise nonessential mitochondrial isoform of phosphoenolpyruvate carboxykinase (PEPCK), which bridges the TCA cycle and glycolysis by converting oxaloacetate into phosphoenolpyruvate (PEP), becomes critical under conditions when glucose import or catabolism are impaired (18). Based on these results, it is assumed that utilization of glucose through glycolysis does not play an essential role under normal conditions (12, 13, 17, 19). This is further supported by the finding that the parasite can tolerate depletion of another glycolytic enzyme, fructose-bisphosphate aldolase (ALD), when glucose was absent (20).

Several lines of evidence, however, suggest that certain reactions associated with glycolysis are needed by tachyzoites for yet-unknown reasons. First, the gluconeogenic enzyme fructose 1,6-bisphosphatase 2 (FBP2) is refractory to gene deletion, and its conditional repression led to altered glycolytic flux concomitant with parasite death even in the presence of glucose (14). These results suggest a vital role of futile cycling between glycolysis and gluconeogenesis for the parasite. Second, deletion of the two lactate dehydrogenases (LDH) almost completely abrogated parasite propagation *in vivo* (21). Further analysis indicates that lactate fermentation is crucial under physiological or hypoxic conditions. The reaction catalyzed by LDH regenerates NAD^+^, which is required for continuous operation of glycolysis (21). Together, these results suggest that glycolysis does play critical roles. In order to get more insights into the physiological significance and mechanisms of central carbon metabolism in *Toxoplasma*, in this study we focused on the biological functions of pyruvate kinase (PYK), which catalyzes the last energy-yielding reaction of glycolysis by converting PEP to pyruvate (22, 23).

T. gondii tachyzoites express two catalytically active PYK enzymes (PYK1 and PYK2) with distinct enzymatic properties (22, 24). The two enzymes also display different subcellular localizations within the parasite (24, 25). PYK1 resides in the cytosol, whereas PYK2 is located primarily in the apicoplast, and possibly also in the mitochondrion (24–26). Cytosolic PYK1 is believed to function in canonical glycolysis to convert PEP into pyruvate, which is then transported into mitochondria and converted to acetyl-coenzyme A (CoA) to drive the TCA cycle (16, 19, 25). PYK2 is supposed to catalyze pyruvate synthesis in the apicoplast, which in turn provides substrates for the synthesis of isoprenoids and fatty acids via the MEP (2-C-methyl-d-erythritol 4-phosphate) and FAS2 (type 2 fatty acid synthesis) pathways, respectively (19, 25, 27–29). In the absence of a known pyruvate transporter in the apicoplast, PYK2 is predicted to be essential because MEP and FAS2 are critical for tachyzoite growth (19, 29).

Here, we performed a systematic genetic and biochemical dissection of the two PYK enzymes in T. gondii, and the results significantly advance our knowledge of carbon metabolism in this parasite. We found that pyruvate serves as a nodal metabolite, connecting pathways located in at least three compartments of the parasite, i.e., cytosol, mitochondrion, and apicoplast. Our data suggest that although the parasite can utilize a repertoire of carbon sources, a sustained supply of pyruvate is vital to parasite growth.

## RESULTS

### PYK1 is critical for the growth of T. gondii tachyzoites.

**FIG 1:**
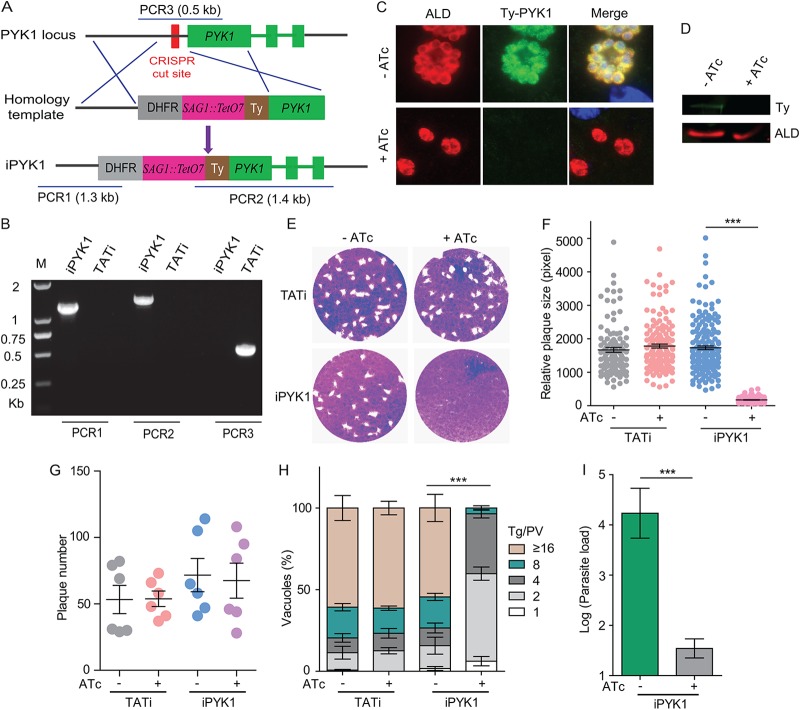
Conditional knockdown of *PYK1* results in severe growth defects. (A) Strategy used to construct the conditional knockdown strain iPYK1, which was done by replacing the endogenous TgPYK1 promoter with a tetracycline regulatable promoter (SAG1::TetO7) in the TATi line. (B) Diagnostic PCR on a representative iPYK1 clone. (C and D) Depletion of *PYK1* expression in iPYK1 by ATc treatment. Lane M, molecular size marker. Intracellular parasites were treated with 0.5 μg/ml ATc for 4 days or left untreated, and subsequently they were subjected to IFA (C) or Western blot (D) analyses using mouse anti-Ty (PYK1 was Ty tagged in iPYK1) and rabbit anti-T. gondii ALD. (E) Plaque assay demonstrating the growth defects of PYK1-depleted mutants. (F and G) Relative sizes (pixel size calculated by Photoshop) (F) and number (G) of plaques from three independent experiments from panel E. ***, *P *< 0.001 by Student's *t* test. (H) Intracellular replication assay comparing parasite proliferation under indicated conditions. TATi and iPYK1 parasites were pretreated with 0.5 μg/ml ATc for 48 h or left untreated. Subsequently they were allowed to infect HFF monolayers for 20 min, and invaded parasites were cultured under corresponding pretreatment conditions for 24 h to determine the number of parasites in each parasitophorous vacuole (PV). Values shown are means ± SEM from three independent experiments (*n* = 3), each with three replicates. ***, *P *< 0.001 by two-way ANOVA. (I) Parasite loads in the peritoneal fluids of nude mice (*n* = 3 for each group) 7 days postinfection. Nude mice were infected with iPYK1 tachyzoites (10^4^/mouse) by intraperitoneal injection. Subsequently they were either left untreated or treated with ATc in drinking water for 7 days, and parasite loads in peritoneal fluids were determined by qPCR. ***, *P *< 0.001 by Student's *t* test.

To analyze the function of PYK1, we first attempted to delete the *PYK1* gene using clustered regularly interspaced short palindromic repeat (CRISPR)/Cas9-assisted homologous gene replacement. However, no viable mutants could be obtained after transgenic selection, indicating a critical function of PYK1. Therefore, we constructed an inducible knockdown strain (iPYK1) to deplete PYK1 expression in an anhydrotetracycline (ATc)-dependent manner by a promoter replacement strategy (Fig. 1A). The ATc-regulatable promoter (pSAG1-TetO7) along with the pyrimethamine selection marker (dihydrofolate reductase [DHFR]) was inserted immediately upstream of the coding sequence of *PYK1* in the TATi strain. A Ty epitope was fused to the N terminus of PYK1 during the construction of iPYK1. Diagnostic PCRs confirmed the correct integration of pSAG1-TetO7 in isolated clonal mutants (Fig. 1B). Treatment of iPYK1 mutant with ATc efficiently shut down the expression of PYK1, as confirmed by the disappearance of Ty signal in immunofluorescence assays (IFA) (Fig. 1C) as well as in immunoblot analysis (Fig. 1D). Phenotypically, the iPYK1 mutant exposed to ATc (off-state) formed miniscule plaques, in contrast to the normal plaques without ATc treatment (on-state) (Fig. 1E and F). The number of plaques seemed similar under both conditions (Fig. 1G). Intracellular replication of the ATc-treated iPYK1 was also notably impaired (Fig. 1H). To check the contribution of PYK1 to parasite growth *in vivo*, the iPYK1 strain was used to infect CD1-Nude mice (immunodeficient, lacking T cells), and parasite loads in mouse peritoneal fluids were determined by quantitative PCR (qPCR) 7 days postinfection. For the infected mice that were treated with ATc to deplete PYK1 expression, parasites in peritoneal fluids were barely detectable and were below the reliable detection limits of qPCR (Fig. 1I). This was in sharp contrast to the control mice that did not receive ATc in drinking water (Fig. 1I). These results show that PYK1 is also crucial for parasite propagation *in vivo*.

To confirm the specificity of the observed phenotypes, we complemented the iPYK1 strain by expressing an hemagglutinin (HA)-tagged PYK1 from the *UPRT* (uracil phosphoribosyl-transferase) locus (Fig. S1A). The complemented strain (iPYK1 comp) was confirmed by diagnostic PCRs (Fig. S1B) and IFA (Fig. S1C). As expected, both parasite growth (Fig. S1D) and replication (Fig. S1E) were fully restored in the PYK1-complemented strain. Interestingly, ectopic expression of PYK2 neither in the cytosol nor in the apicoplast rescued the growth defects of PYK1 depletion mutants (Fig. S1C, S1F, and S1G), suggesting distinct physiological roles of the two enzyme isoforms.

10.1128/mBio.00898-19.1 FIG S1 *PYK1* complementation fully restored the growth defects of *PYK1* depletion mutants. (A) Schematic diagram showing the insertion of a *PYK1*-expressing minigene into the *UPRT* locus of the iPYK1 strain by CRISPR/Cas9-mediated site-specific integration. (B) Diagnostic PCRs on a selected iPYK1comp clone. (C) Expression of the complementing PYK1, full-length PYK2 (apico), or truncated PYK2 that localized to the cytosol (cyto) from the *UPRT* locus, as shown by IFA using mouse anti-HA (complementing PYK1/PYK2 was HA tagged) and rabbit anti-TgALD. (D to G) Plaque (D and F) and intracellular replication assays (E and G) comparing the growth and proliferation of PYK1 depletion strain before and after PYK1 (D and E) or PYK2 (F and G) complementation. Values shown are means ± SEM from three independent experiments (*n* = 3), each with three replicates. ***, *P < *0.001 by two-way ANOVA. PYK2 (apico) was the full-length PYK2, whereas PYK2 (cyto) lacked the N-terminal apicoplast targeting sequence. Download FIG S1, TIF file, 6.1 MB. Copyright © 2019 Xia et al. 2019 Xia et al. This content is distributed under the terms of the Creative Commons Attribution 4.0 International license.

Inactivation of certain glycolytic enzymes is known to cause the accumulation of intermediates toxic to cells, such as fructose 1,6-bisphosphate (FBP) in ALD-deficient mutants (20, 30). Such toxicity could be relieved by omission of glucose from the culture medium (20, 30). To examine whether accumulation of toxic intermediates is responsible for growth defects in the *PYK1* depletion mutants, the iPYK1 strain was cultured in media with or without glucose and replication rates were compared. For the parental strain TATi, ATc treatment did not affect parasite proliferation but removal of glucose significantly slowed down replication, confirming the known importance of glucose (Fig. S2A). In contrast, ATc treatment on iPYK1 drastically reduced its replication, which could not be reversed by removal of glucose (Fig. S2A). Omission of glutamine further decreased the replication rates of PYK1-depleted mutants (Fig. S2B). These results therefore exclude accumulation of toxic intermediates as a cause of the impaired growth in PYK1-depleted mutants.

10.1128/mBio.00898-19.2 FIG S2 Removal of glutamine but not glucose further exacerbated the growth defects of PYK1-depleted mutants. TATi or iPYK1 parasites were left untreated or pretreated with ATc for 48 h, and subsequently they were subjected to intracellular replication analysis by growth in glucose-replete versus -depleted (A) or glutamine-replete versus -depleted (B) conditions for 24 h. Values are means ± SEM from three independent experiments (*n* = 3), each with three replicates. **, *P < *0.01; ***, *P < *0.001; both by two-way ANOVA. Download FIG S2, TIF file, 1.4 MB. Copyright © 2019 Xia et al. 2019 Xia et al. This content is distributed under the terms of the Creative Commons Attribution 4.0 International license.

### Knockdown of PYK1 alters the levels of cellular ATP and pertinent metabolites.

**FIG 2:**
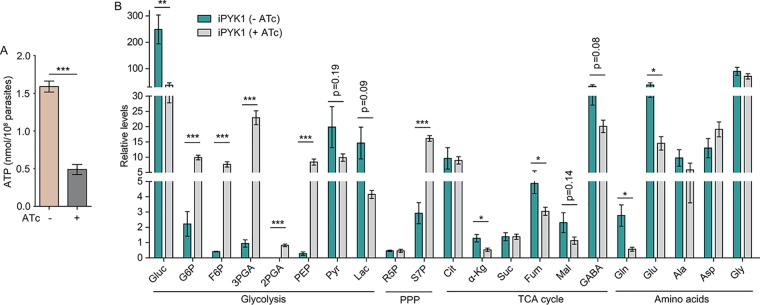
*PYK1* depletion leads to reduced ATP and global alteration of metabolite levels. The iPYK1 strain was left untreated or treated with ATc for 48 h. Subsequently the parasites were purified and lysed for ATP (A) and metabolite level (B) measurements. Means ± standard errors of the means (SEM) from three (A) or four (B) independent experiments were graphed. *, *P < *0.05; **, *P < *0.01; ***, *P < *0.001; all by Student's *t* test. PPP, pentose phosphate pathway.

We next examined the ATP levels in the iPYK1 mutant. Fresh extracellular parasites derived from ATc-treated cultures showed 60 to 70% reduction in ATP (Fig. 2A). To test whether PYK1 depletion affected intermediates of the central carbon metabolism, the iPYK1 strain was cultured with or without ATc for 2 days, followed by assessment of selected metabolites by gas chromatography-mass spectrometry (GC-MS) or liquid chromatography-MS (LC-MS). We selected parasites exposed to ATc for 2 days because the PYK1 enzymatic activity was reduced by 75% (from 126.8 μmol/min/mg lysate at day 0 to 31.2 μmol/min/mg lysate at day 2). In addition, longer ATc treatments did not yield sufficient parasites for metabolic analysis due to the growth arrest imposed by PYK1 depletion. Nonetheless, ATc treatment for 2 days resulted in a significantly higher abundance of most glycolytic metabolites upstream of pyruvate (such as glucose-6-phosphate [G6P], fructose 6-phosphate [F6P], and PEP), as well as the pentose phosphate pathway (PPP) intermediate sedoheptulose 7-phosphate (S7P) (Fig. 2B). Consistent with the catalytic activity of PYK1, levels of pyruvate and lactate were reduced after PYK1 knockdown (Fig. 2B). Several TCA cycle intermediates were also reduced in PYK1-depleted parasites (Fig. 2B). We also observed a decline in glutamine and glutamic acid after repression of PYK1 (Fig. 2B). These results suggest a central homeostatic role of PYK1 in ATP synthesis and carbon metabolism of T. gondii.

### PYK1 depletion impairs the metabolic flux of [^13^C]glucose and [^13^C]glutamine.

**FIG 3:**
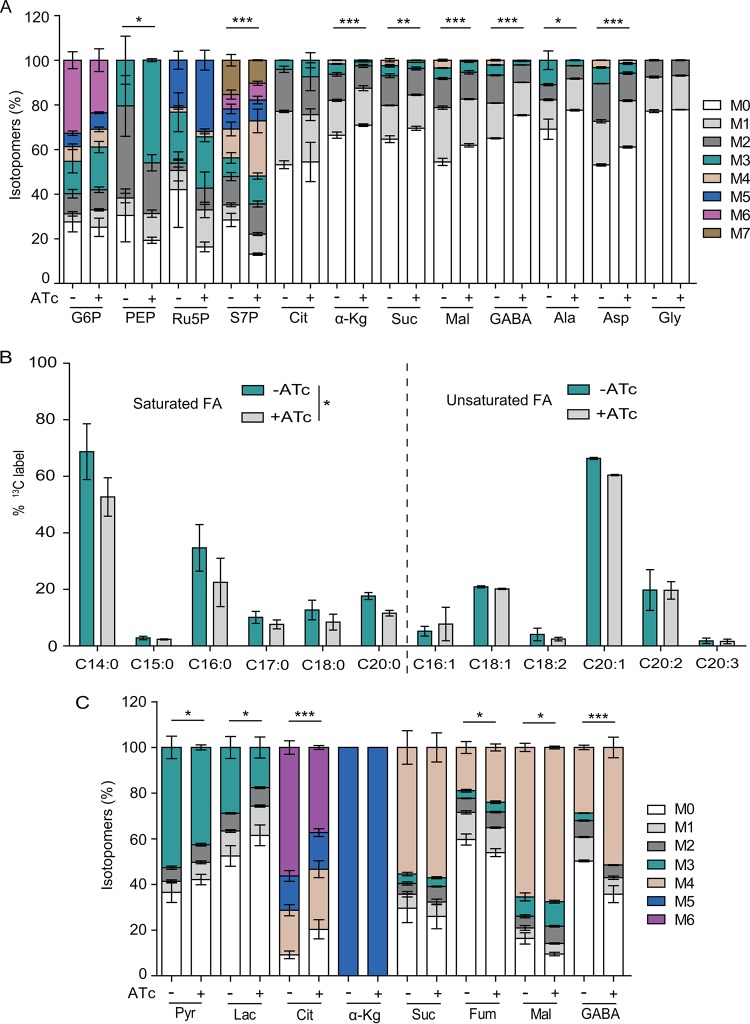
PYK1 is required for the incorporation of glucose- or glutamine-derived carbon into glycolysis, TCA cycle, amino acids, and fatty acid synthesis pathways. (A) Freshly egressed tachyzoites (2.5 × 10^7^) of iPYK1 left untreated or pretreated with ATc for 48 h were collected, purified, and then incubated in medium containing 8 mM [U-^13^C]glucose for 4 h. Incorporation of ^13^C into sugar phosphates, TCA cycle intermediates, and amino acids was determined by LC-MS or GC-MS. Values are means ± SEM from three independent experiments (*n* = 3). *, *P* < 0.05; **, *P* < 0.01; ***, *P* < 0.001; all by two-way ANOVA. (B) Intracellular tachyzoites were grown in media containing 8 mM [U-^13^C]glucose with or without ATc for 2 days, and incorporation of ^13^C into fatty acids (FA) was analyzed by GC-MS. Values are means ± SEM from three independent experiments (*n* = 3). *, *P < *0.05 by Wilcoxon rank-sum test. (C) Incorporation of glutamine-derived carbon into glycolysis and TCA cycle intermediates. Metabolic labeling was done as described for panel A but using 8 mM [U-^13^C]glutamine instead of [U-^13^C]glucose. Values are means ± SEM from three independent experiments (*n* = 3). *, *P <* 0.05; ***, *P* < 0.001; both by two-way ANOVA.

To validate the metabolic alterations in PYK1-depleted parasites, we determined the carbon flux in the iPYK1 mutant using ^13^C-labeled glucose. The tracer labeling of fresh extracellular parasites showed that inclusion of glucose-derived ^13^C into PEP and S7P was significantly increased upon ATc-mediated depletion of PYK1 (Fig. 3A), which is consistent with the increased relative abundance of these two metabolites in corresponding cultures (Fig. 2B), as well as with the aforementioned reduction in PYK activity in the parasite lysate. However, changes in the incorporation of ^13^C into other glycolytic intermediates (such as G6P and F6P) were not as obvious. As expected, flux of ^13^C into most TCA cycle intermediates were reduced (Fig. 3A). We also observed a decline in labeling of alanine and aspartate (Fig. 3A), which likely reflect an impaired pyruvate synthesis and TCA cycle, respectively.

To evaluate the impact of PYK1 depletion on fatty acid synthesis, intracellular parasites of the iPYK1 strain were cultured in medium supplemented with [^13^C]glucose in the presence or absence of ATc for 2 days. Subsequently, the parasites were harvested, purified, and subjected to GC-MS analysis. Conditional knockdown of PYK1 led to a notable decrease in the incorporation of ^13^C into saturated fatty acids, including myristic acid (C_14:0_), palmitic acid (C_16:0_), stearic acid (C_18:0_), and arachidonic acid (C_20:0_) (Fig. 3B). In contrast, labeling of unsaturated fatty acids (C_16:1_, C_18:1_, C_18:2_, C_20:1_, C_20:2_, and C_20:3_) was not much influenced (Fig. 3B). Because saturated fatty acids shorter than C18 are produced primarily in the apicoplast (31), our data suggest that perturbation of cytosolic PYK1 impacts *de novo* fatty acid synthesis in the apicoplast.

Under normal conditions, either glucose or glutamine is sufficient for parasite growth and survival. However, depletion of PYK1 arrested the lytic cycle even when glutamine was available (Fig. 1E to H), which suggested efficient glutamine utilization also requires PYK1. To examine this notion, metabolic labeling of extracellular parasites was performed using [^13^C]glutamine. Upon PYK1 depletion, flux of glutamine-derived carbon into TCA cycle intermediates (fumarate and malate) and gamma-aminobutryic acid (GABA) was significantly increased (Fig. 3C), which is consistent with increased consumption of glutamine under this condition. However, incorporation of glutamine-derived carbon into citrate was greatly reduced (Fig. 3C), suggesting impaired operation of the TCA cycle due to reduced supply of acetyl-CoA in the mitochondrion following PYK1 depletion. Consistent with this, we also observed reduced carbon flux into pyruvate and lactate (Fig. 3C). These data indicate that efficient operation of the TCA cycle using glutamine as a carbon source depends on PYK1.

### PYK1-deficient parasites accumulate amylopectin.

**FIG 4:**
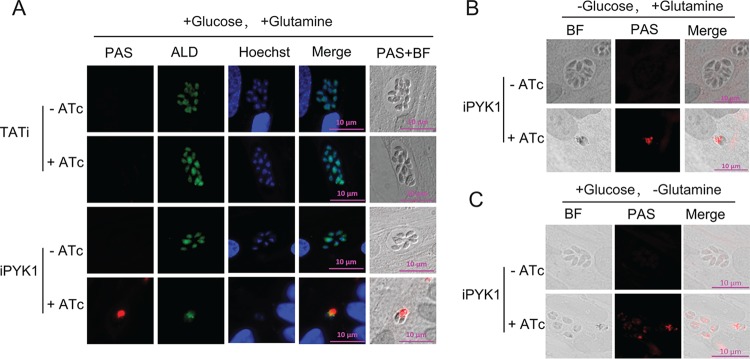
Amylopectin accumulation in *PYK1*-depleted parasites determined by PAS staining. Tachyzoites of the TATi or iPYK1 line were pretreated under the indicated conditions (with or without 0.5 μg/ml ATc, with or without 4,500 mg/liter glucose, with or without 8 mM glutamine) for 2 days. The parasites then were forced to egress and infect fresh host monolayers. Invaded parasites were allowed to grow for another 24 h under corresponding pretreatment conditions. Subsequently cells were fixed and subjected to IFA analysis with rabbit anti-T. gondii ALD. After IFA, PAS staining procedures were used to stain amylopectin. BF, bright field.

Examination of PYK1-depleted parasites by phase-contrast microscopy revealed an aberrant morphology. Most of them had one or more bright spots within the parasite or within the residual body space of the parasitophorous vacuole (Fig. S3, yellow arrow), which appeared similar to the starch granules in other cells and amylopectin granules in *Toxoplasma* bradyzoites (32). To confirm our notion, parasites were subjected to periodic acid-Schiff (PAS) staining for polysaccharides. Indeed, ATc-treated iPYK1 mutant was strongly stained in red, which was barely detectable in control cultures (TATi or iPYK1 without ATc) (Fig. 4A). To discern the carbon source for amylopectin accumulation, the iPYK1 strain was cultured in medium lacking either glucose or glutamine. Surprisingly, amylopectin staining remained equally significant with either carbon source (Fig. 4B and C). Removal of both carbon sources was not possible, because host cells did not survive long enough to perform this test.

10.1128/mBio.00898-19.3 FIG S3 Accumulation of granular structures (yellow arrow) in ATc-treated iPYK1 parasites. The iPYK1 strain was cultured in medium with (+) or without (−) ATc for three days and then directly imaged under a phase-contrast microscope. Download FIG S3, TIF file, 5.0 MB. Copyright © 2019 Xia et al. 2019 Xia et al. This content is distributed under the terms of the Creative Commons Attribution 4.0 International license.

To further examine the contribution of glutamine or glucose to amylopectin formation, we generated a double mutant (iPYK1-*Δgt1*) lacking the glucose transporter GT1 in the iPYK1 strain (Fig. S4A and B). The iPYK1-*Δgt1* mutant showed notable amylopectin staining in the presence of ATc (Fig. S4C) despite its inability to import host-derived glucose. However, when glutamine was omitted, PAS staining was no longer apparent (Fig. S4C). Therefore, the only condition to alleviate amylopectin accumulation in the iPYK1 mutant was to simultaneously block the parasites’ access to both nutrients. These data show that both glucose and glutamine can be utilized as carbon sources for amylopectin synthesis and reveal the vital importance of cytosolic pyruvate kinase in maintaining metabolic homeostasis.

10.1128/mBio.00898-19.4 FIG S4 Potential carbon sources for amylopectin accumulation in PYK1-depleted mutants. (A) Schematic illustration of deleting *GT1* in iPYK1 to obtain the iPYK1-*Δgt1* mutant. *GT1* was replaced by the chloramphenicol-resistant minigene *CAT* through CRISPR/Cas9-mediated homologous recombination. (B) Diagnostic PCRs on a selected iPYK1-*Δgt1* clone. (C) PAS staining to detect amylopectin accumulation. Tachyzoites of the iPYK1-*Δgt1* strain were first pretreated under the indicated conditions for 2 days and then used to infect fresh HFF monolayers. Invaded parasites were cultured under corresponding pretreatment conditions for another 24 h and then subjected to PAS staining. Download FIG S4, TIF file, 6.1 MB. Copyright © 2019 Xia et al. 2019 Xia et al. This content is distributed under the terms of the Creative Commons Attribution 4.0 International license.

### Lactate and alanine can partially rescue the growth defect of PYK1-depleted mutants.

**FIG 5:**
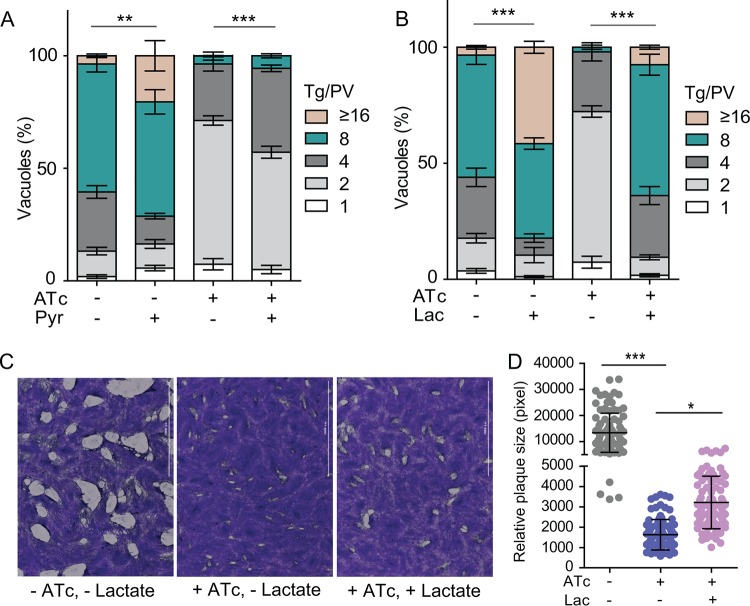
Lactate partially restores the growth defects of *PYK1*-depleted mutants. (A and B) The iPYK1 strain was pretreated under the indicated conditions (with or without 0.5 μg/ml ATc, without glucose, with 8 mM glutamine, with or without 8 mM pyruvate, with or without 8 mM lactate) for 2 days. Subsequently they were allowed to infect fresh HFF cells and grown for another 24 h under corresponding pretreatment conditions. The number of parasites in each PV then was determined. (C) A 7-day plaque assay of the iPYK1 strain cultured under indicated conditions. (D) Relative size of plaques in panel C. Values are means ± SEM from over 90 plaques for each strain. *, *P *< 0.05; **, *P *< 0.01; ***, *P *< 0.001; both by one-way ANOVA with Bonferroni posttests.

PYK1 catalyzes the production of pyruvate, and its knockdown resulted in decreased levels of pyruvate. To investigate whether pyruvate supplementation can rescue the growth defect in PYK1-depleted parasites, levels of intracellular replication under different culture conditions (with or without ATc, with or without pyruvate, with glutamine, and without glucose) were compared. Pyruvate supplementation slightly increased the replication rates of iPYK1 both in the presence and absence of ATc, as judged by a higher proportion of bigger vacuoles (Fig. 5A). A lack of more profound improvement by pyruvate may be due to poor uptake of this nutrient. We also examined lactate, which can be converted to pyruvate by LDH. Indeed, irrespective of ATc treatment, lactate enhanced parasite reproduction even better than pyruvate (Fig. 5B). This finding was further endorsed by plaque assays, which showed that the PYK1-depleted mutant formed significantly larger plaques in the presence of exogenous lactate (Fig. 5C and D). Improvement of parasite growth by lactate was also confirmed in the type 2 strain ME49, which replicated significantly faster in the presence of lactate when glucose was not available (Fig. S5A). Interestingly, enhancement of ME49 growth by lactate did not occur in the absence of LDH (Fig. S5A). Likewise, rescue of PYK1-depleted mutants by lactate also strictly depended on LDH1 (Fig. S5B to E), suggesting that lactate must be converted to pyruvate to stimulate parasite growth.

10.1128/mBio.00898-19.5 FIG S5 Improvement of parasite growth by lactate requires lactate dehydrogenase (LDH). (A) Intracellular replication for the ME49 and ME49 *Δldh1 Δldh2* strains, which were grown in medium (without glucose, with glutamine) with or without 8 mM lactate. The number of parasites in each PV was determined 24 h after infection. Values are means ± SEM from three independent experiments (*n* = 3), and over 100 vacuoles were counted for each sample. ***, *P < *0.001 by two-way ANOVA. (B) Diagram of *LDH1* deletion in iPYK1 by CRISPR/Cas9-mediated homologous gene replacement. (C) Diagnostic PCR on an iPYK1-*Δldh1* clone. (D) Plaque assays on iPYK1 and iPYK1-*Δldh1* strains under conditions with or without 0.5 μg/ml ATc or 8 mM lactate. (E) Intracellular replication assay of iPYK1 and iPYK1-*Δldh1* strains under the indicated conditions, as done in Fig. 5. Values are means ± SEM from three independent experiments (*n* = 3). ***, *P < *0.001 by two-way ANOVA. Download FIG S5, TIF file, 5.1 MB. Copyright © 2019 Xia et al. 2019 Xia et al. This content is distributed under the terms of the Creative Commons Attribution 4.0 International license.

In similar assays, lactate also improved the growth of the iPYK1-*Δgt1* strain (which is unable to import glucose from host cells) irrespective of ATc treatment (Fig. S6A). Alanine was also able to increase the replication rates of the iPYK1-*Δgt1* strain (Fig. S6B), although not as efficiently as lactate. For comparison, acetate supplementation modestly improved the replication of the iPYK1-*Δgt1* strain in the absence of ATc (Fig. S6C), likely due to its ability to rescue the defects caused by deletion of GT1, as reported before (13). However, acetate did not influence the proliferation of the iPYK1-*Δgt1* strain in the presence of ATc (Fig. S6C), indicating that acetate could not rescue the impairment imposed by PYK1 depletion. Collectively, these results show that lactate and alanine, but not acetate, can serve as substitute nutrients in the PYK1 knockdown mutant.

10.1128/mBio.00898-19.6 FIG S6 Alanine but not acetate improved the growth of *PYK1*-depleted mutants. The iPYK1-*Δgt1* strain was first pretreated under the indicated conditions and then subjected to intracellular replication analysis (replication time was 24 h for panel A and 48 h for panels B and C) under corresponding pretreatment conditions. Values are means ± SEM from three independent experiments (*n* = 3). *, *P < *0.05; ***, *P < *0.001; both by two-way ANOVA. Download FIG S6, TIF file, 1.2 MB. Copyright © 2019 Xia et al. 2019 Xia et al. This content is distributed under the terms of the Creative Commons Attribution 4.0 International license.

### PYK2 located in the apicoplast is dispensable for lytic cycle.

**FIG 6:**
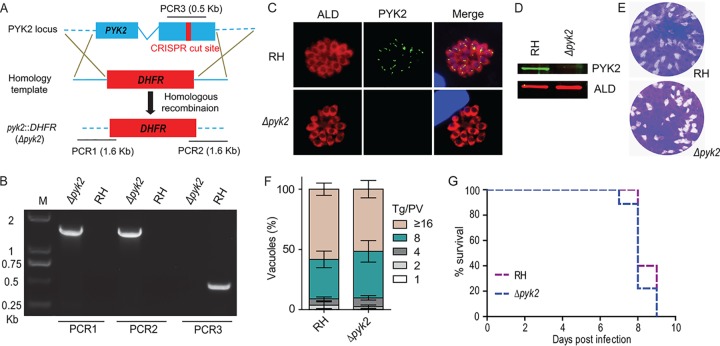
Generation and characterization of a *PYK2* deletion mutant. (A) Schematic illustration of knocking out *PYK2* by CRISPR/Cas9-mediated homologous gene replacement in the RH *Δhxgprt* strain (RH). (B) Diagnostic PCRs on a *Δpyk2* clone. (C and D) IFA (C) and Western blotting (D) confirming the disruption of *PYK2*, using mouse anti-PYK2 and rabbit anti-TgALD. (E) A 7-day plaque assay comparing the overall growth of the *PYK2* deletion mutant to that of parental strain RH. (F) Intracellular replication assay checking parasite proliferation *in vitro*. Invaded parasites were allowed to replicate for 24 h, and subsequently the number of parasites in each PV was determined by IFA. Over 150 vacuoles were analyzed for each strain, and the experiment was repeated three times independently (*n* = 3). (G) Survival curves of mice infected with tachyzoites of indicated strains. The wild-type (WT) and *Δpyk2* mutants were used to infect ICR mice (100 tachyzoites/mouse, *n* = 10 mice for each strain) by intraperitoneal injection, and the survival of mice was monitored daily.

We next studied the physiological roles of PYK2. CRISPR/Cas9-assisted gene replacement was used to delete *PYK2* in the RH strain (Fig. 6A). Surprisingly, the *Δpyk2* mutant could be readily obtained despite its predicted essentiality. PCR, IFA, and immunoblot assays confirmed successful ablation of the *PYK2* locus in isolated clones (Fig. 6B to D). As shown in the plaque and replication assays, the growth of the *Δpyk2* mutant was indistinguishable from that of the parental strain (Fig. 6E and F). In addition, PYK2 was also found to be dispensable for parasite virulence in the mouse infection model (Fig. 6G). These results demonstrate that PYK2 located in the apicoplast is not required for parasite survival, growth, or virulence, which is in stark contrast to the current model of carbon metabolism in the apicoplast.

### Simultaneous inactivation of PYK1 and PYK2 abrogates tachyzoite growth.

**FIG 7:**
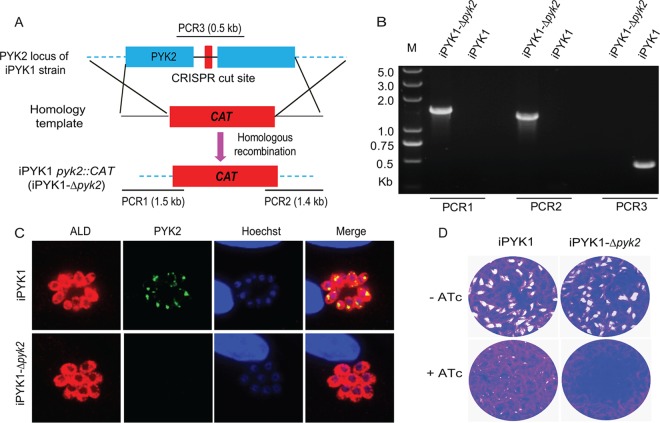
Inactivation of both *PYK1* and *PYK2* completely blocks parasite growth. (A) Schematic illustration of deleting *PYK2* in iPYK1 to produce the iPYK1-*Δpyk2* double mutant. (B) Diagnostic PCRs on a selected iPYK1-*Δpyk2* clone. (C) IFA analysis confirming the loss of *PYK2* expression in the iPYK1-*Δpyk2* mutant. (D) Plaque assay comparing the growth of the iPYK1-*Δpyk2* mutant to that of iPYK1 with or without 0.5 μg/ml ATc treatment.

Because the parasite harbors two PYK enzymes, we reasoned a contribution of PYK2 to the residual growth in the PYK1-depleted mutant. To investigate this, we constructed an iPYK1-*Δpyk2* double mutant, in which *PYK2* was replaced by the *CAT* (chloramphenicol acetyltransferase) selection marker in the iPYK1 strain (Fig. 7A). PCR screening confirmed the occurrence of double homologous recombination at the *PYK2* locus, as intended (Fig. 7B). Immunostaining verified the loss of PYK2 protein in the iPYK1-*Δpyk2* strain (Fig. 7C). Exposure of the iPYK1-*Δpyk2* mutant to ATc in plaque assays completely blocked parasite growth, which contrasted with small plaques in the iPYK1 strain treated with ATc (Fig. 7D). Inability of the iPYK1-*Δpyk2* mutant to grow in the presence of ATc was also apparent in routine cultures, where only a few misshaped parasites could be detected after 5 days of ATc treatment. Akin to *PYK1*-depleted iPYK1 mutant, the growth defect of the double mutant was also partially rescued by lactate (Fig. S7). These results indicate an evident, albeit dispensable, role of PYK2 during the lytic cycle and confirm lactate as an auxiliary carbon source for T. gondii.

10.1128/mBio.00898-19.7 FIG S7 Partial rescue of *PYK1* and *PYK2* double-deficient mutants by lactate. Parasites were pretreated under the indicated conditions (with or without 0.5 μg/ml ATc, with or without 4,500 mg/liter glucose, with 8 mM glutamine, with or without 8 mM lactate) for 2 days. They were then used to infect fresh HFF cells and grown for another 24 h under corresponding pretreatment conditions. Subsequently the number of parasites in each PV was determined by IFA. Values are means ± SEM from two independent experiments (*n* = 2), each with three replicates. ***, *P < *0.001 by two-way ANOVA. Download FIG S7, TIF file, 0.8 MB. Copyright © 2019 Xia et al. 2019 Xia et al. This content is distributed under the terms of the Creative Commons Attribution 4.0 International license.

### Deficiency in pyruvate kinases coincides with a loss of apicoplasts.

**FIG 8:**
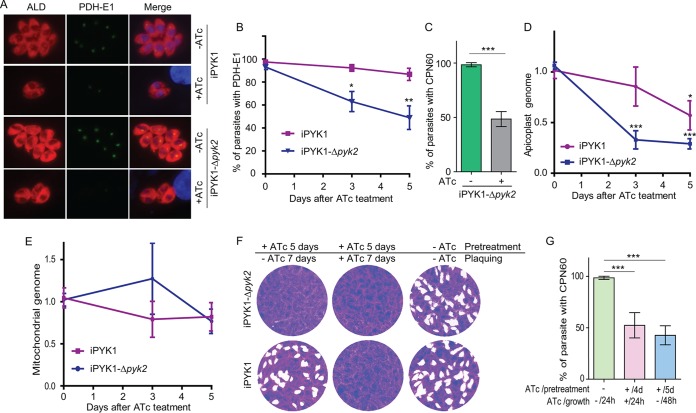
PYK inactivation leads to loss of apicoplast. (A) IFA checking the localization of the apicoplast-targeted enzyme PDH-E1α. Parasites were left untreated or treated with ATc for 5 days and then fixed to stain with rabbit anti-TgALD and mouse anti-PDH-E1α, which were visualized by Alexa-594- and -488-conjugated secondary antibodies, respectively. (B) Percentage of parasites with apicoplast-localized PDH-E1α over the course of ATc treatment. Values are means ± SEM from more than 100 parasites for each data point, and experiments were repeated three times independently. *, *P* < 0.05; **, *P* < 0.01; both by Student's *t* test. (C) Similar to panel B, but the iPYK1-*Δpyk2* mutants were first treated with ATc for 5 days and then stained for another apicoplast marker, CPN60. (D and E) Relative abundance of apicoplast and mitochondrial DNA in parasites treated with ATc for 0, 3, or 5 days. Each treatment was repeated three times independently (*n* = 3), and each sample was done with two technical repeats. Values are means ± SEM. *, *P < *0.05; **, *P < *0.01; ***, *P < *0.001; all by Student's *t* test. (F) Reversibility of the growth defects caused by PYK1 depletion. Indicated parasites were left untreated or were treated with ATc for 5 days (pretreatment), and then they were subjected to plaque assay with or without ATc for 7 days (plaquing). (G) Reversibility of apicoplast loss in the iPYK1-*Δpyk2* mutants. Parasites were left untreated or were treated with ATc for 4 or 5 days (pretreatment), and then they were used to infect fresh HFF cells and grown with or without ATc for 24 or 48 h (growth) for IFA analysis to determine the frequency of CPN60-expressing parasites. Values are means ± SEM from three independent experiments. ***, *P < *0.001 by Student's *t* test.

Pyruvate is a key metabolic precursor in the apicoplast because it is the substrate for FAS2 and MEP pathways located in this organelle (19, 29). Therefore, we tested whether the integrity of apicoplast was affected when pyruvate supply was restricted. To do this, the iPYK1 and iPYK1-*Δpyk2* strains were grown in the presence or absence of ATc, and then the expression of the PDH-E1α subunit, a known marker of the apicoplast (25, 33), was determined. Under control conditions, nearly all parasites in both strains exhibited a bright signal for PDH-E1α (Fig. 8A) in the apicoplast. Normal PDH-E1α staining in the iPYK1-*Δpyk2* strain without ATc treatment (Fig. 8A) suggested that PYK2 did not have a significant role in maintaining the apicoplast under normal conditions. Exposure of iPYK1 mutant to ATc caused an apparent reduction in the signal intensity, although the percentage of parasites with visible PDH-E1α did not change significantly (Fig. 8B). On the other hand, ATc treatment of the iPYK1-*Δpyk2* strain for 3 and 5 days led to a complete loss of PDH-E1α (Fig. 8A) staining in 30% and 50% tachyzoites, respectively (Fig. 8B). Similar results were also obtained with another apicoplast protein, CPN60 (Fig. 8C). To further examine the impact of PYK inactivation on apicoplast biogenesis, quantitative PCR was used to assess the DNA contents in this organelle, using the nuclear genome as a reference and the mitochondrial genome for comparison. Indeed, the iPYK1-*Δpyk2* strain exhibited a significant decrease in the apicoplast DNA upon ATc treatment (Fig. 8D). Consistent with the PDH-E1α and CPN60 staining, the loss of apicoplast DNA in ATc-treated iPYK1 mutant was not as severe as that in the double mutant (Fig. 8D), indicating that pyruvate production by PYK2 becomes critical to maintain the apicoplast when PYK1 is not expressed. As expected, no significant change in the mitochondrial genome of either the iPYK1 or iPYK1-*Δpyk2* strain was observed irrespective of ATc treatment (Fig. 8E). Taken together, these data show a vital role of pyruvate kinase enzymes in apicoplast maintenance. To check whether the growth inhibition and loss of apicoplast caused by PYK depletion/deletion are reversible, the iPYK1 or iPYK1-*Δpyk2* strain was first pretreated with ATc for 4 to 5 days to achieve significant reduction in growth and apicoplast biogenesis. Subsequently, they were subjected to plaque assays and apicoplast integrity assessment (by analyzing CPN60 expression) with or without ATc. Removal of ATc treatment efficiently restored the growth of ATc-treated iPYK1 but not the iPYK1-*Δpyk2* strain (Fig. 8F). Similarly, apicoplast loss in the iPYK1-*Δpyk2* strain could also not be reversed by ATc removal (Fig. 8G). Together, these results suggest that inactivating PYK1 alone arrests parasite growth and does not result in parasite death. In contrast, simultaneous inactivation of both PYK1 and PYK2 is synthetically lethal.

## DISCUSSION

Metabolic plasticity in carbon metabolism is considered crucial for successful parasitism of T. gondii in diverse organisms and host cell types (10, 12, 13). Several studies have reported that the parasite can utilize glucose as well as glutamine as the major carbon resources (10, 12, 13). Either one of the two nutrients is sufficient for parasite survival, and several proteins involved in glucose acquisition and glycolysis appear to be dispensable provided glutaminolysis remains intact in the parasite (10, 12, 13). Here, we show that the last step of glycolysis catalyzed by pyruvate kinase is critical for the survival of T. gondii tachyzoites irrespective of the two nutrients. The parasite expresses two pyruvate kinases with distinct catalytic properties and subcellular locations (25, 26). It was predicted that PYK1 serves the canonical glycolysis in the cytosol, while PYK2 produces pyruvate from glycolysis-derived PEP in the apicoplast (19). Pyruvate in turn is used for the likely essential isoprenoids and fatty acid synthesis pathways in this organelle (19). Moreover, the GTP/ATP-yielding reaction of PYK2 may also provide energy to the essential biosynthetic processes in apicoplast (19). Thus, PYK2 was anticipated to be essential for parasite survival. Unexpectedly, however, our study demonstrates a dispensable nature of PYK2 and crucial functions of PYK1 in standard cultures replete with glucose and glutamine.

**FIG 9:**
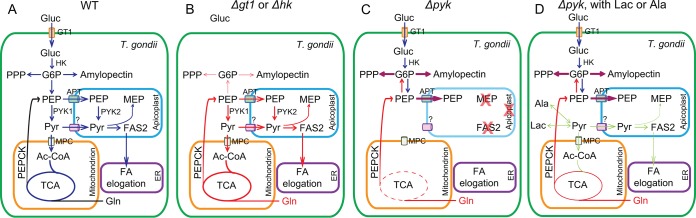
Pyruvate is at the center of T. gondii carbon metabolism. (A) WT parasites have full capacity to use glucose and glutamine as carbon sources, and glucose is preferred under optimal conditions. (B) Mutants lacking GT1 or HK cannot use host-derived glucose; therefore, they heavily rely on glutamine for survival. Glutamine can be used to fuel the TCA cycle for energy production. It is also used to replenish PEP and other glycolytic intermediates through PEPCK and gluconeogenesis. (C) In *Δpyk* mutants (*pyk1*^−^ *pyk2^−^*), glucose imported from host cells cannot be fully catabolized, leading to accumulation of glycolytic and PPP intermediates as well as amylopectin. These mutants cannot efficiently use glutamine either. Inability to convert PEP to pyruvate blocks the full operation of the TCA cycle (dashed red circle) because of reduced acetyl-CoA (Ac-CoA) production. Deficiency in pyruvate supply also compromises metabolic activities (MEP and FAS2) in apicoplasts that require pyruvate as substrates, resulting in loss of this organelle. Consequently, their growth is completely arrested. (D) The growth of PYK-deficient mutants is partially restored by lactate or alanine supplementation, which can be converted to pyruvate in the parasites. The question mark denotes the to-be-identified pyruvate transporter in apicoplast membranes.

Comparison of the PYK mutants generated here with GT1 and HK deletion mutants published elsewhere offers significant new insights into carbon metabolism of T. gondii (12, 17). The latter two strains survive by inducing glutaminolysis and activation of gluconeogenesis to meet their bioenergetic needs (Fig. 9A and B) (12, 13, 17). In contrast, the PYK1-deficient mutant barely grows even in the presence of glutamine and intact mitochondrial PEPCK (PEPCK_mt_) (18), which can be explained as follows. The mitochondrial acetyl-CoA, derived from pyruvate by the action of BCKDH, enables a canonical TCA cycle in the mitochondrion (16). In the absence of GT1 or HK, sufficient acetyl-CoA can still be made from glutamine-derived oxaloacetate, which is converted to pyruvate via the catalytic action of PEPCK_mt_ and PYK1, respectively (12, 13, 17, 18). This supply of pyruvate is apparently disrupted in the iPYK1 mutant, leading to impaired acetyl-CoA synthesis in the mitochondrion and eventual inhibition of the TCA cycle (Fig. 9C). Hence, efficient utilization of glutamine, not just glucose, also requires PYK1 (Fig. 5B and Fig. S6A, compare the first and third columns; ATc treatment drastically reduced the growth of iPYK1 cultured in glutamine). Our [^13^C]glutamine labeling experiments clearly confirm this notion (Fig. 3C). Consistent with this, the ATP level was significantly reduced in the PYK1-depleted parasites (Fig. 2A), even more so than what is reported in the *Δgt1* and *Δhk* mutants (13, 17).

We also observed accumulation of amylopectin in the PYK1-deficient strains (Fig. 4), a phenomenon not yet reported in the *Δgt1* or *Δhk* mutants (12, 13, 17). Amylopectin staining was equally significant with glucose or glutamine as the carbon source, suggesting a severe disruption of carbon homeostasis. Again, this is probably caused by the inability of PYK1-depleted parasites to completely break down glucose or glutamine through the TCA cycle (Fig. 9C). In contrast, *Δgt1* or *Δhk* mutants use glutamine as the major carbon source, much of which is likely used via the TCA cycle to generate ATP (12, 13, 17). Therefore, little glutamine would be used to synthesize amylopectin (Fig. 9B) (12, 13, 17). Nonetheless, polysaccharide accumulation is probably not the main reason underlying the poor growth of PYK1-deficient parasites, because it was also obvious in the iPYK1 strain rescued by lactate (Fig. 9D). Hence, it appears that carbon metabolism is more severely impaired in the PYK1 mutant than that in the *Δgt1* or *Δhk* strains due to disrupted catabolism of glucose and glutamine (12, 13, 17). Our results show that inability to make pyruvate from glucose- or glutamine-derived PEP has more drastic consequences than the inability to utilize the sugar or amino acid *per se*. In accord with this premise, growth of the iPYK1-deficient strain was reinstated by carbon sources that can be converted to pyruvate, such as lactate or alanine (Fig. 5 and Fig. S6), and the rescue by lactate was strictly dependent on LDH1 to convert it to pyruvate (Fig. S5). Pyruvate itself does not rescue the iPYK1 mutant as efficiently (Fig. 5A), likely due to its poor import, which is consistent with previous data from the *Δgt1* strain (12). In this sense, it would be interesting to know how lactate and alanine are imported. The recently described formate-nitrite transporter (FNT) family proteins are top candidates due to their confirmed lactate transport activity (34). Our data show unprecedented metabolic plasticity that enables the parasite to utilize lactate and alanine besides glucose and glutamine as additional carbon sources. This metabolic flexibility resembles that of tumor cells, which preferentially use glucose and glycolysis (Warburg effect). However, recent studies clearly show that tumors also use nonglucose nutrients to fulfill their metabolic needs (35). For example, human non-small-cell lung cancer cells effectively take up lactate and use it in the TCA cycle (36). Similarly, pancreatic ductal adenocarcinoma (PDAC) cells stimulate pancreatic stellate cells to secrete alanine, which outcompetes glucose or glutamine to fuel the TCA cycle in PDAC (37).

Another difference between the *Δgt1* and PYK1-deficient strains is their lipid biogenesis. The *Δgt1* mutant displays modest reduction in major phospholipids due to impaired synthesis of very long acyl chains (e.g., C_26:1_) (13). Consequently, the mild growth and lipid synthesis defects in the *Δgt1* mutant could be reversed by acetate, which is likely converted to acetyl-CoA via acetyl-CoA synthetase and thereby supports elongation of fatty acids (13). PYK1-deficient mutants also exhibited an impaired synthesis of long acyl chains (C_14:0_ to C_20:0_) (Fig. 3B), which are probably made by FAS2 in the apicoplast. One possible reason for reduced FAS2 in this mutant is decreased supply of glycolysis-derived pyruvate to the apicoplast. Limitation of pyruvate in this organelle may also interfere with the MEP pathway. Indeed, depletion of PYK1 led to declined PDH-E1α expression and loss of apicoplast, which was accentuated in the double mutants lacking both PYK1 and PYK2. Surprisingly, the *Δpyk2* mutants grow normally, implying that supply of pyruvate is not significantly affected, likely due to metabolite import from the cytosol. Therefore, it seems quite plausible that the apicoplast membrane harbors a transporter to access a glycolysis-derived pool of pyruvate (Fig. 9). Likewise, the data endorse PYK1 as the main enzyme meeting the pan-organellar requirement of pyruvate in the mitochondrion as well as in the cytosol and apicoplast. On the other hand, PYK2 has a minor role under normal conditions but becomes somewhat important to keep the parasites viable when PYK1 is depleted. It can therefore be argued that a sustained pyruvate homeostasis is critical to the utilization of multiple nutrients and metabolic flexibility in the carbon metabolism of T. gondii.

A dispensable role of PYK2 in tachyzoites is rather enigmatic and raises a number of questions. Absence of noticeable growth defects in the *Δpyk2* mutant indicates alternative ways to acquire pyruvate and energy in the apicoplast. Indeed, there is another ATP-yielding enzyme localized in the apicoplast, phosphoglycerate kinase 2 (PGK2), which can potentially compensate for the loss of PYK2 (although PYK2 prefers to generate GTP over ATP), at least in terms of energy (25). In addition, there may be transporters in the apicoplast membrane to import ATP/GTP and possibly other nucleotides needed to replicate its genome. Interestingly, PGK2 is absent from the related parasite *Plasmodium* (19, 27, 28); hence, PYK2 is the only known energy-producing enzyme in its apicoplast, indicating an essential function (19, 27, 28). Not surprisingly, the data from genome-wide genetic screens do confirm this notion (38, 39).

## MATERIALS AND METHODS

### Biological resources.

The RH-*Δhxgprt* (RH) and TATi strains were propagated in human foreskin fibroblast (HFF) cells (purchased from the ATCC, Manassas, VA, USA) as described before (40). All other transgenic lines were constructed from these two strains and are described in more detail below. Anhydrotetracycline (ATc) (TaKaRa Bio USA, Inc., Mountain View, CA, USA) at a final concentration of 0.5 μg/ml was used to deplete *PYK1* expression in all iPYK1-based strains.

### Construction of plasmids and genetically modified strains.

All primers and plasmids used in this study are listed in Tables S1 and S2 in the supplemental material, respectively. Locus-specific CRISPR plasmids were generated by replacing the *UPRT* targeting guide RNA (gRNA) in pSAG1-Cas9-sg*UPRT* with corresponding gRNAs, using site-directed mutagenesis as described previously (40, 41). Other plasmids were constructed by multifragment ligation using the ClonExpress II one-step cloning kit (Vazyme Biotech, Nanjing, China). All transgenic strains were constructed by CRISPR/Cas9-mediated site-specific gene editing as previously described (40, 41). Detailed procedures for the construction of plasmids and strains are provided in the supplemental material (Text S1). Other techniques commonly used to determine protein expression and parasite growth, such as IFA, plaque assay, intracellular replication assay, and ATP measurement were performed as previously described (13, 20, 21). More details are also provided in the supplemental material (Text S1).

10.1128/mBio.00898-19.8 TEXT S1 Supplementary methods. Download Text S1, DOCX file, 0.03 MB. Copyright © 2019 Xia et al. 2019 Xia et al. This content is distributed under the terms of the Creative Commons Attribution 4.0 International license.

10.1128/mBio.00898-19.9 TABLE S1 Primers used in this study. Download Table S1, XLSX file, 0.01 MB. Copyright © 2019 Xia et al. 2019 Xia et al. This content is distributed under the terms of the Creative Commons Attribution 4.0 International license.

10.1128/mBio.00898-19.10 TABLE S2 (2-1) Plasmids used in this study. (2-2) Transgenic parasites used in this study. Download Table S2, DOCX file, 0.02 MB. Copyright © 2019 Xia et al. 2019 Xia et al. This content is distributed under the terms of the Creative Commons Attribution 4.0 International license.

### PAS staining (IFA compatible).

Parasites (TATi, iPYK1, or iPYK1-*Δgt1*) were first pretreated under the indicated conditions (with or without 0.5 μg/ml ATc, with or without 4,500 mg/liter glucose, and with or without 8 mM glutamine) for 2 days. The parasites then were forced to egress by needle passage and allowed to invade fresh HFF monolayers seeded on coverslips for 1 h. After washing off extracellular parasites, invaded ones were cultured under corresponding pretreatment conditions for another 24 h. The samples then were fixed with 4% paraformaldehyde, permeabilized with 0.1% Triton X-100, blocked with 10% fetal bovine serum, and then incubated with rabbit anti-ALD for 20 min at room temperature. After extensive washing with phosphate-buffered saline (PBS), Alexa Fluor 488-conjugated goat-rabbit IgG (Life Technologies, Inc., MD, USA) was added and incubated for another 20 min, and we subsequently counterstained the nuclei with Hoechst solution. After immunostaining, modified periodic acid-Schiff (PAS) staining was performed as previously described (42). Briefly, samples were treated with periodic acid solution (Sigma-Aldrich, St. Louis, MO, USA) for 5 min, washed with PBS, and then incubated in Schiff’s reagent (Sigma-Aldrich, St. Louis, MO, USA) for 15 min at room temperature. Subsequently samples were washed with PBS and treated with Mayer’s hematoxylin solution (Beijing Solarbio Science & Technology Co., Ltd., Beijing, China) for 2 min. After extensive washing, samples were visualized under the Olympus BX53 microscope (Olympus Life Science, Tokyo, Japan).

### Metabolomic analysis.

Gas chromatography-mass spectrometry (GC-MS) and ultra-performance liquid chromatography-mass spectrometry (UPLC-MS) were used to determine the relative abundance of selected metabolites in parasite extracts, using [^13^C6-^15^N-L]isoleucine as an internal normalization reference, as described previously (21). Detailed protocols are provided in the supplemental material (Text S1).

### Virulence test in mice.

Seven-week-old female ICR mice were purchased from the Hubei provincial Center of Disease Control and Prevention. They were maintained under conditions specified by the Administration of Affairs Concerning Experimental Animals. The animal experiments were approved by the ethical committee of Huazhong Agricultural University (permit number MO-2016-055). Freshly purified tachyzoites were used to infect 7-week-old female ICR mice by intraperitoneal injection (10 mice per strain, 100 tachyzoites per mouse in a volume of 200 μl serum-free medium). The survival and symptoms of mice then were monitored daily.

### qPCR.

Tachyzoites (iPYK1 or iPYK1-*Δpyk2* strain) with or without 3 or 5 days’ ATc treatment were forced to egress by needle passage and purified for DNA extraction using the EasyPure genomic DNA kit (Transgen Biotech, Beijing, China). Thirty ng DNA from each sample was subject to qPCR analysis, using primers (listed in Table S1) designed to amplify fragments from the apicoplast genome (*EF-Tu*), mitochondrial genome (*CytB*), and nuclear genome (*UPRT*), respectively, as previously described (43–45). The qPCR was performed using the Power SYBR green PCR master mix (Toyobo Co., Ltd., Osaka, Japan), and all reactions were performed on the ABI ViiA 7 detection system (Life Technologies, Inc., Rockville, MD, USA). The threshold cycle (*C_T_*) values for the nuclear genome amplification were used as a normalization reference to compare apicoplast and mitochondrial genome abundance across samples. The 2^ΔΔ^*^CT^* method was used to estimate the changes of apicoplast and mitochondrial genome abundance after ATc treatment. Every sample was independently tested three times, each with two technical replicates.

### Statistical analysis.

Statistical comparisons were performed in Prism 5 (GraphPad Software, Inc., CA, USA) using Student's *t* tests, Wilcoxon rank-sum test, one-way analysis of variance (ANOVA) with Bonferroni posttests, or two-way ANOVA as indicated in the figure legends.
