# Supplementary figures and images for: Pyruvate Homeostasis as a Determinant of Parasite Growth and Metabolic Plasticity in Toxoplasma gondii
Source: mBio. 2019 Jun 11;10(3):e00898-19. doi: 10.1128/mBio.00898-19 (PMC6561023; doi:10.1128/mBio.00898-19)

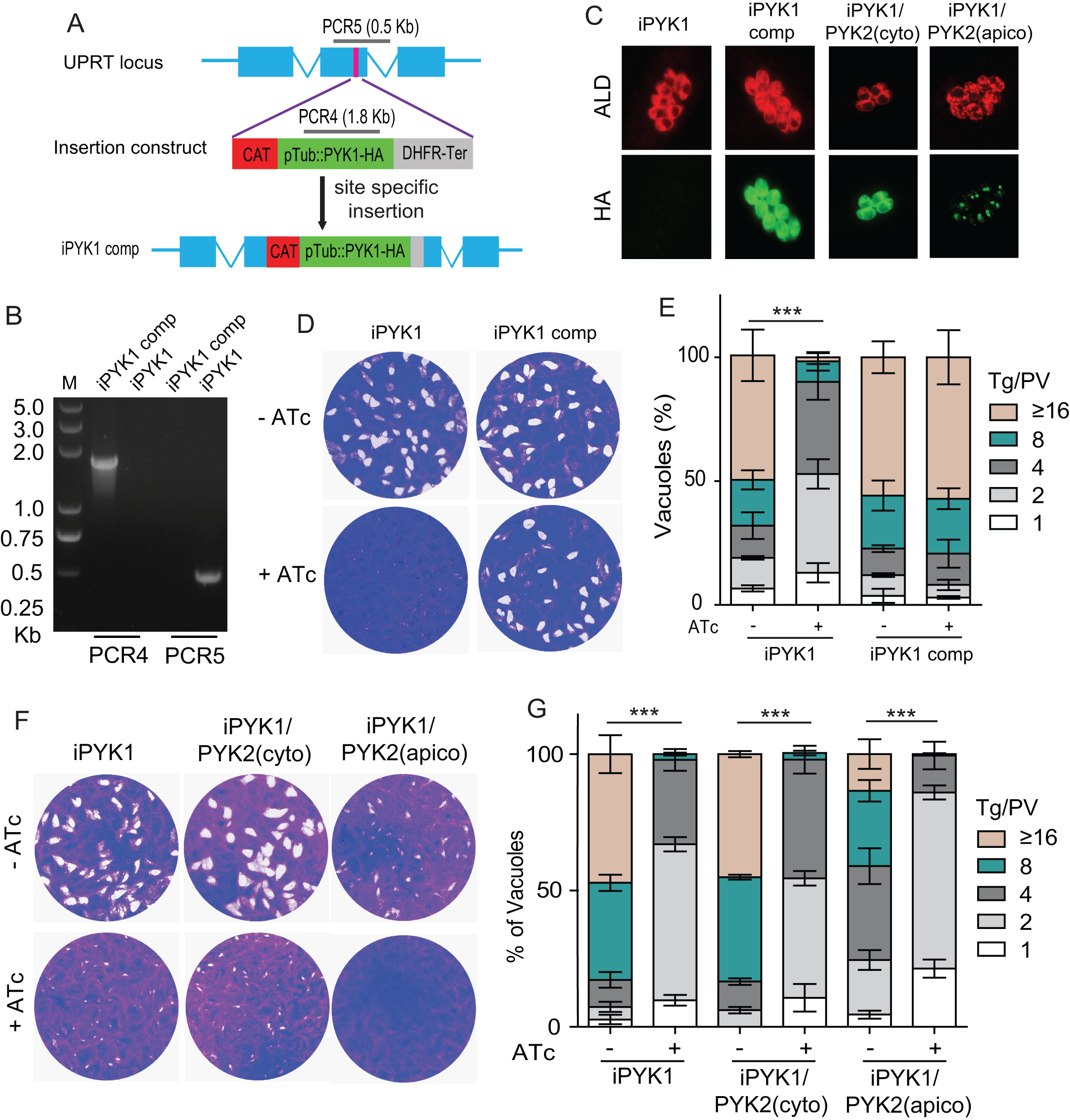

Supplement: FIG S1 [file mBio.00898-19-sf001.tif]

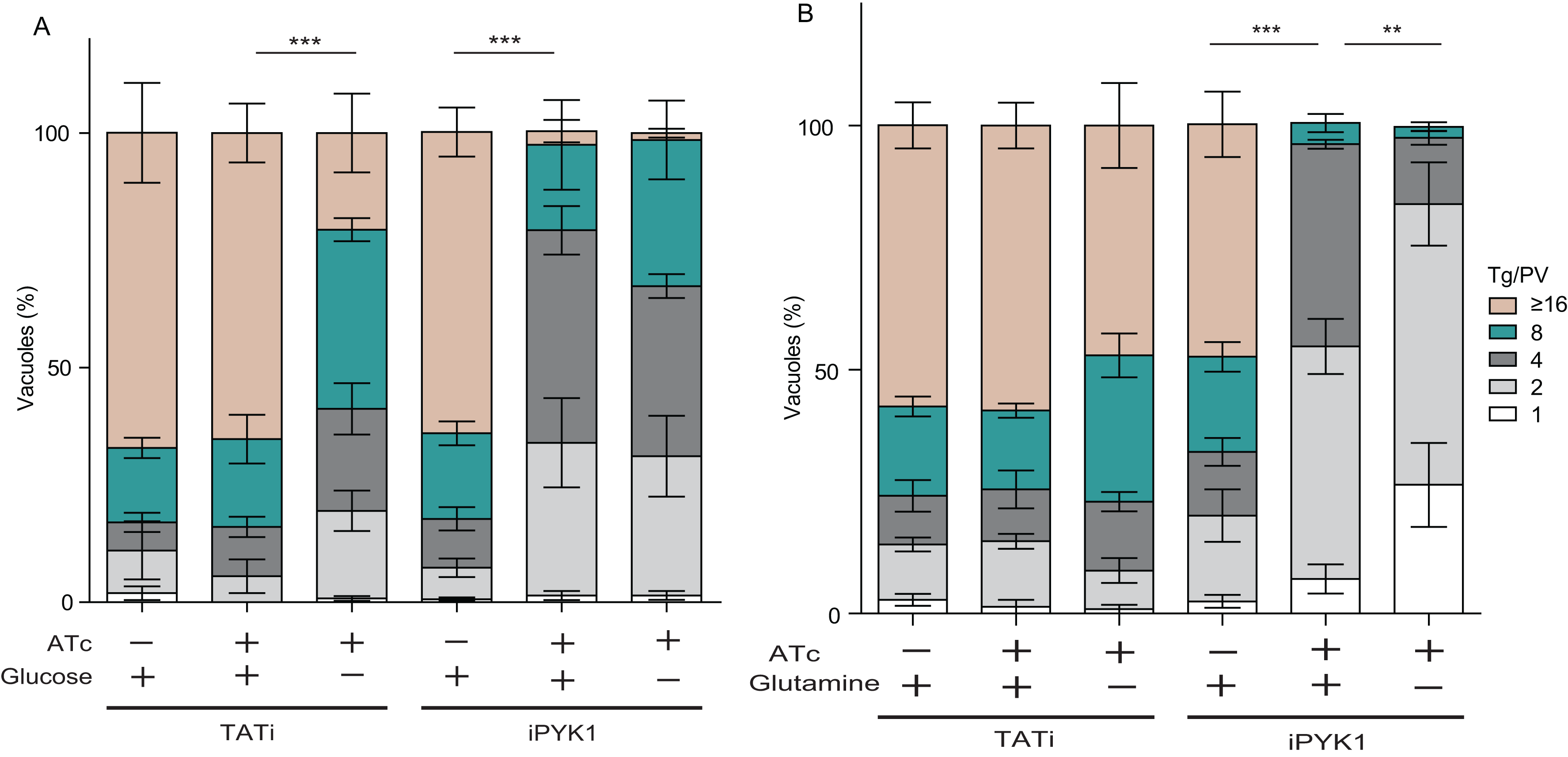

Supplement: FIG S2 [file mBio.00898-19-sf002.tif]

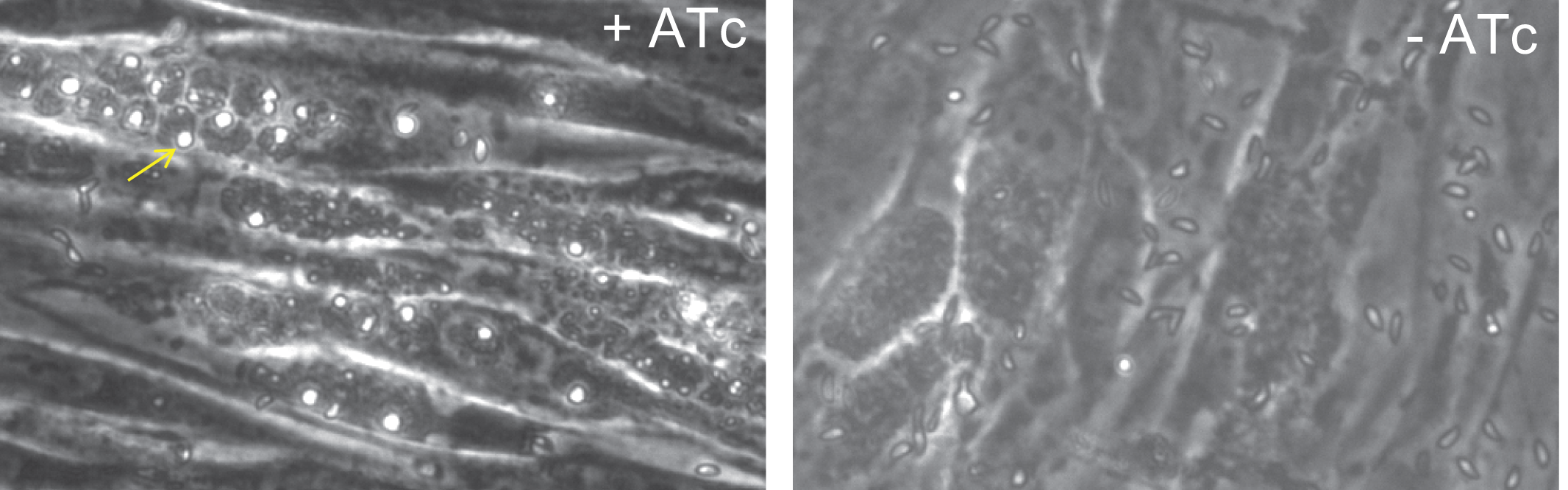

Supplement: FIG S3 [file mBio.00898-19-sf003.tif]

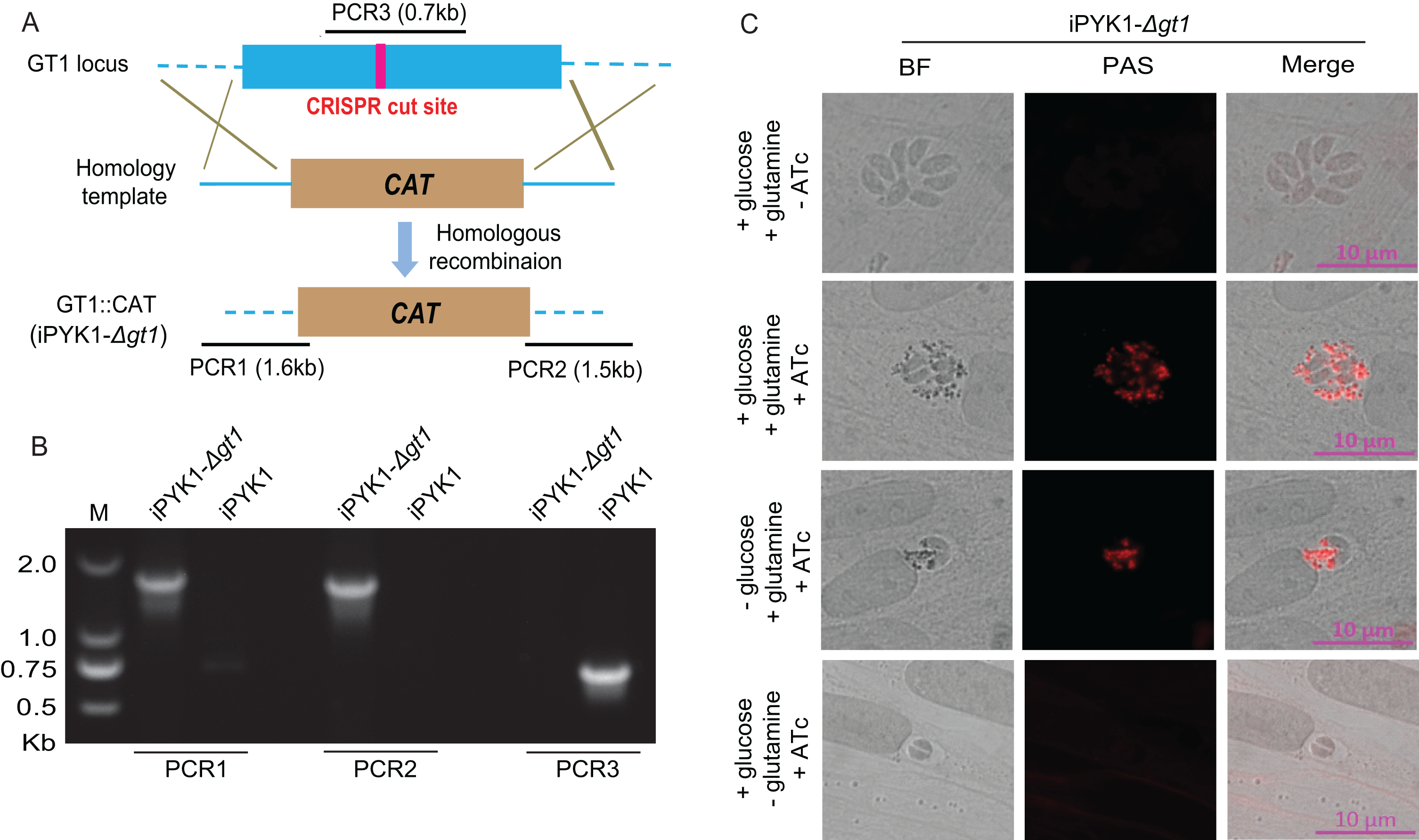

Supplement: FIG S4 [file mBio.00898-19-sf004.tif]

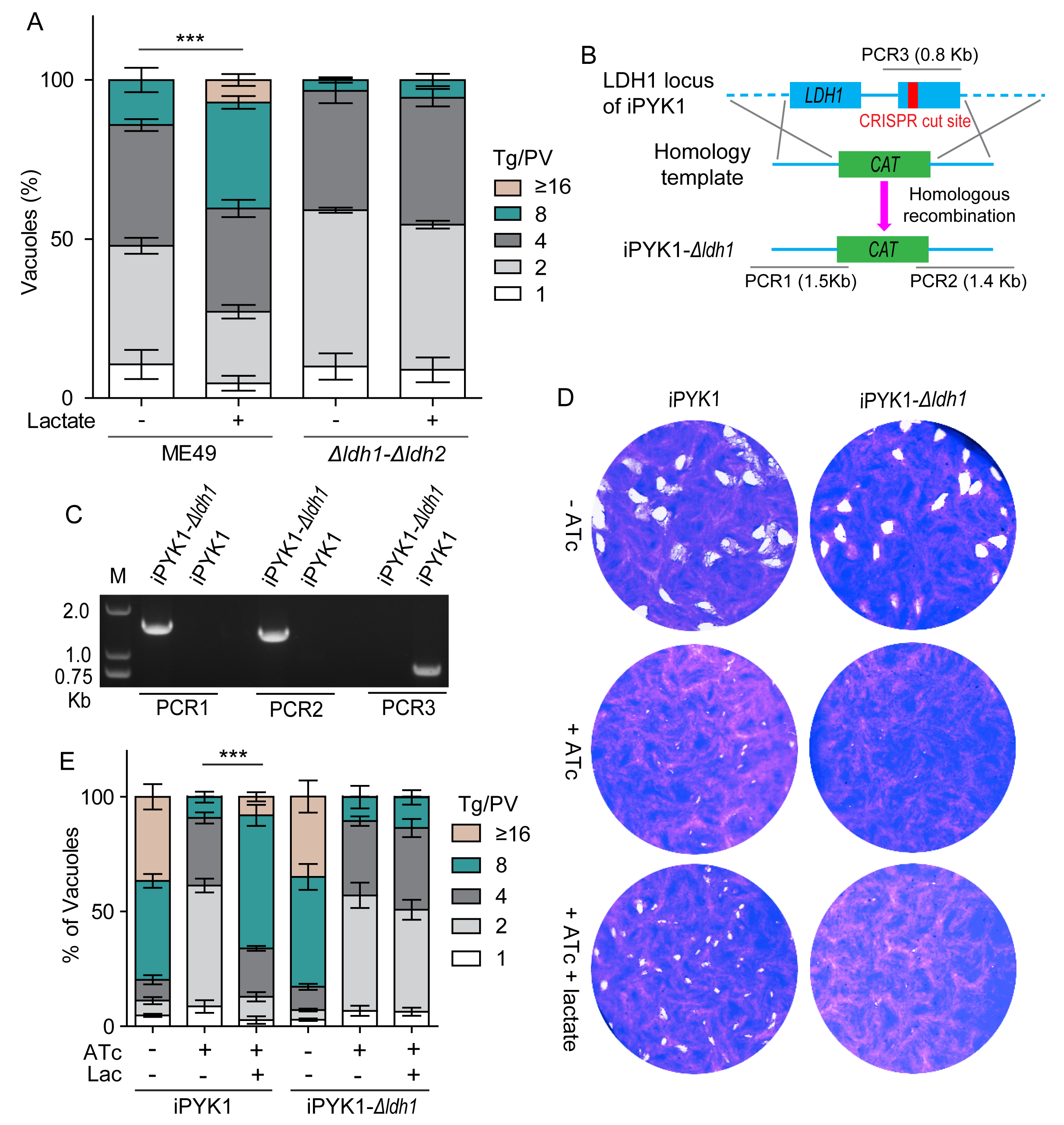

Supplement: FIG S5 [file mBio.00898-19-sf005.tif]

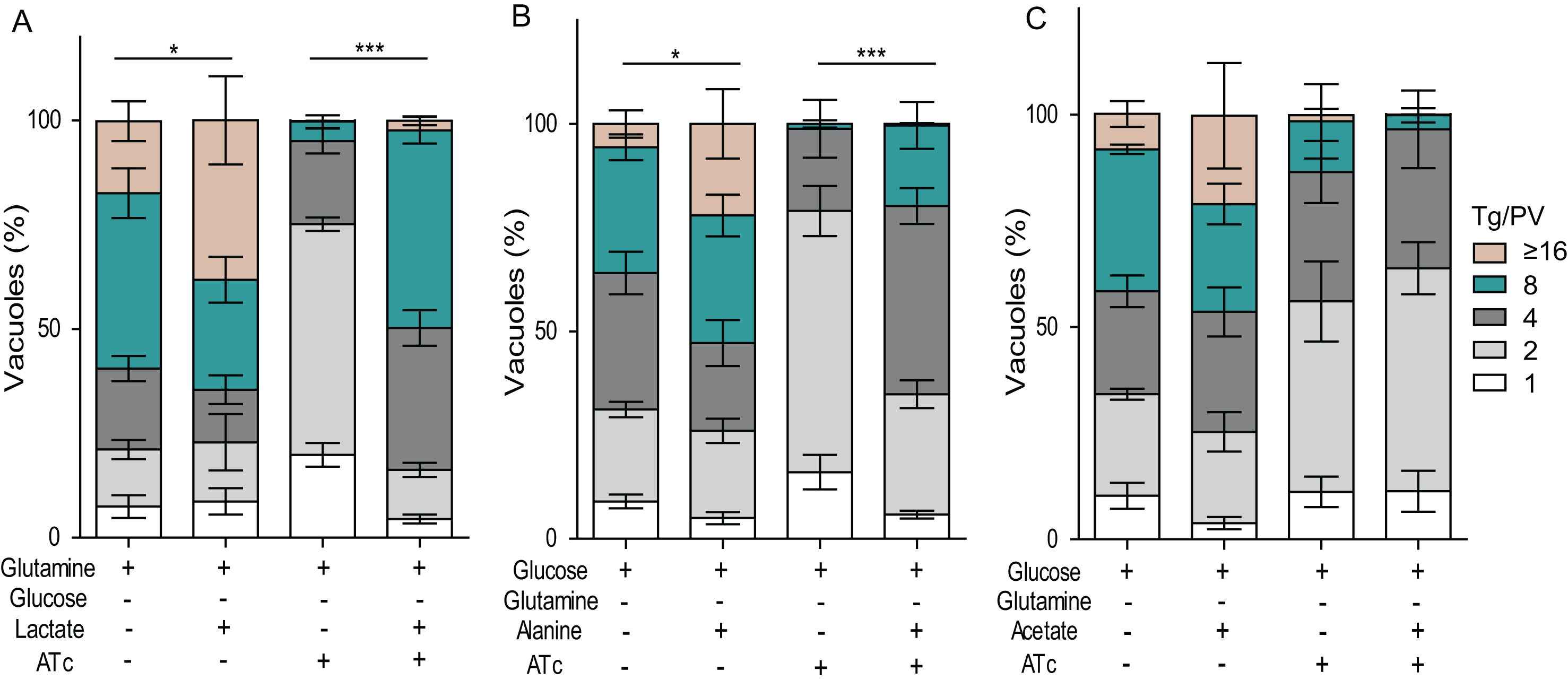

Supplement: FIG S6 [file mBio.00898-19-sf006.tif]

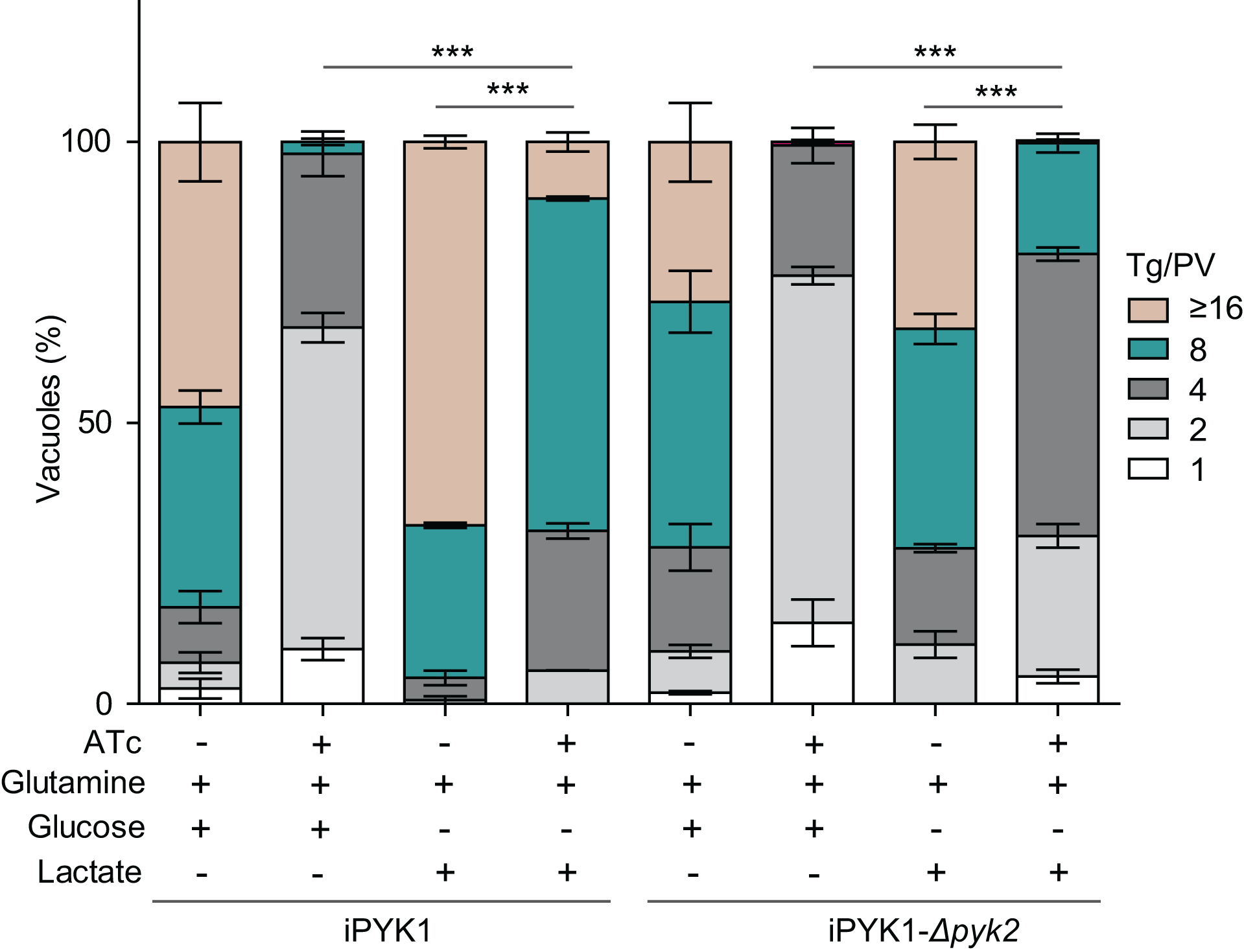

Supplement: FIG S7 [file mBio.00898-19-sf007.tif]
