# Supplementary material for: Pyruvate Homeostasis as a Determinant of Parasite Growth and Metabolic Plasticity in Toxoplasma gondii
Source: mBio. 2019 Jun 11;10(3):e00898-19. doi: 10.1128/mBio.00898-19 (PMC6561023; doi:10.1128/mBio.00898-19)
Supplement: TEXT S1 [file mBio.00898-19-s0001.docx]

**Supplementary Methods**

**Plasmid construction**

Plasmids pPYK1-*DHFR*, pPYK2-*DHFR*, pPYK2-*CAT* and pGT1-*CAT* used as homology templates to replace *PYK1*, *PYK2* or *GT1* with selection markers *DHFR* or *CAT* were made as following: the 5'- and 3'-UTRs (about 1 Kb) of *PYK1, PYK2* or *GT1*, as well as the *DHFR* or *CAT* mini genes were PCR amplified and cloned into pUC19 using the ClonExpress II One Step Cloning Kit (Vazyme Biotech, Nanjing, China). Primers used to amplify each fragment were listed in Table S1 and genomic DNA of RH was used as template for amplification of 5'- and 3'-UTRs. *DHFR* and *CAT* were amplified from pUPRT-*DHFR*-D and pLDH2-*CAT* respectively.

The plasmid pTet-off::PYK1-Ty was constructed by cloning the first 1 Kb of *PYK1* genomic sequence (starting from ATG), as well as the *DHFR*-TetO7 amplified from p7TetOS1 plasmid (gift from the L. David Sibley lab at Washington University in St Louis) into pUC19::5H (amplified from pPYK1-*DHFR*) through the One Step Cloning Kit (Vazyme Biotech, Nanjing, China). It was used as the homologous template to replace the *PYK1* promoter with *DHFR*::TetO7.

The *PYK1* complementing plasmid pTub-PYK1::HA-*CAT* was constructed by replacing LDH1 in pTub-LDH1-*CAT* with PYK1-HA, which was amplified from the cDNA of RH. Plasmids pTub-PYK2(apico)-CAT and pTub-PYK2(ctyo)-CAT were constructed in similar ways, which express full length and truncated (AA 358-988) PYK2 respectively. All plasmids were verified by DNA sequencing before use.

**Construction of genetically modified strains**

To construct the iPYK1 mutant, the TetO7-PYK1 amplicon was co-transfected with pSAG1-CAS9-sgPYK1-cKO into TATi. Transfectants were selected with 1 μM pyrimethamine (Sigma Aldrich, St. Louis, MO, USA), cloned by limiting dilution, and identiﬁed by diagnostic PCRs (primers listed in Table S1). The PYK1 complementing strain iPYK1 comp was generated by inserting the amplicon of Tub-PYK1-*CAT* into the *UPRT* locus of iPYK1. Briefly, Tub-PYK1-*CAT* and pSAG1-CAS9-sg*UPRT* were co-transfected into iPYK1, and selected with 30 μM chloramphenicol and 10 μM FUDR. RH *Δpyk2* was generated by replacing *PYK2* with *DHFR* in RH *Δhxgprt*, using CRISPR/CAS9 directed homologous gene replacement described previously (1). The homology template (PYK2-*DHFR*) was PCR amplified from plasmid pPYK2-*DHFR*, and then co-transfected with the CRISPR plasmid pSAG1-CAS9-sgPYK2 into freshly purified RH tachyzoites. Other direct knockout strains were constructed in similar ways. The iPYK1-*Δpyk2* mutant was obtained by replacing *PYK2* with *CAT* in iPYK1, and selecting with 30 μM chloramphenicol (Sigma Aldrich, St. Louis, MO, USA). Similarly, iPYK1-*Δgt1* and iPYK1-*Δldh1* were obtained by replacing *GT1* or *LDH1* with *CAT* in iPYK1 respectively. All transgenic strains were analyzed by diagnostic PCRs and IFA before use.

**Immunofluorescence microscopy**

IFA analyses were performed as previously described (2). The following primary antibodies were used: mouse anti-Ty monoclonal antibody (gifts from the L. David Sibley lab at Washington University School of Medicine), mouse anti-HA (medical & biological laboratories Co., Ltd, Nagoya, Japan), mouse anti PYK2, mouse anti-PDH-E1α, rabbit anti-CPN60 (gift from Dr. Honglin Jia at Harbin Veterinary Research Institute in China) and rabbit anti-ALD (gift from the L. David Sibley lab at Washington University School of Medicine). Primary antibodies were detected by Alexa Fluor 488-conjugated goat anti-mouse IgG or Alexa Fluor 594-conjugated goat anti-rabbit IgG (Life Technologies, Inc., MD, USA) secondary antibodies. Fluorescence images were acquired using the Olympus BX53 microscope (Olympus Life Science, Tokyo, Japan) equipped with an AxioCam 503 mono camera (Zeiss, Gottingen, Germany).

**Plaque assays**

Freshly egressed parasites were purified using 3 μm polycarbonate membrane (GE Healthcare, Little Chalfont, England). The purified parasites were used to infect HFF monolayers seeded on 6-well plates (200 tachyzoites/well, three wells per strain) and grown under indicated conditions for 7 days at 37 °C with 5% CO_2_. Parasites were fixed with 4% paraformaldehyde, stained with crystal violet and imaged as previously described (3).

**Intracellular replication assay**

Parasites pretreated with ± 0.5 μg/ml ATc, ± 4500 mg/L glucose, ± 8 mM glutamine, ± 8 mM lactate, ± 8 mM pyruvate, ± 8 mM alanine, ± 2 mM acetate for 2 days were forced to egress by needle passage. Then they were used to infect fresh HFF cells seeded on coverslips for 20 min. Wash with DMEM medium ((Life Technologies, Inc., Grand island, NY, USA) to remove non-invaded parasites and grew the invaded ones in corresponding pretreatment conditions for 24 or 48 hours. Cells were then ﬁxed and stained (with anti-TgALD) as previously described (2) to determine the number of parasites in each parasitophorous vacuole. A minimum of 150 vacuoles were examined for each sample in each test. All strains were tested three times independently.

**ATP level measurement**

The iPYK1 strain were cultured in media with or without 0.5 μg/ml ATc for two days. Then freshly egressed parasites were filtered using 3 μm polycarbonate membranes and washed 3 times in ice-cold PBS. Subsequently 3×10^7^ parasites were suspended in 100 μl lysis buffer, boiled for 5 min, and then cooled on ice, and then centrifuged (12,000 g, 5 min, 4 °C) to collect the supernatants, as previously described (4). The ATP levels in the supernatant were tested using a commercial ATP colorimetric assay kit (Sigma Aldrich, St. Louis, MO, USA).

**Metabolomic analysis**

To determine the relative abundance of metabolic intermediates in iPYK1 before and after ATc treatment, tachyzoites (3×10^7^) with or without 2-days’ ATc treatment were forced to egress by needle passage, purified by 3 μm membrane filtration, and lysed for metabolite extraction by 50% methanol. Metabolites were measured by GC-MS, using the added compound ^13^C_6_-^15^N-Lisoleucine as internal normalization reference, as described previously (2). Each sample was prepared and analyzed four times independently.

To monitor the incorporation of glucose or glutamine derived carbons into different metabolites in iPYK1, parasites were first grown in regular medium with or without ATc treatment for about two days until natural egress. Subsequently they were collected, washed with glucose free DMEM medium, and incubated in glucose free DMEM supplemented with 8 mM ^13^C_6_-glucose or ^13^C_5_-glutamine (with or without 0.5 μg/ml ATc, according to corresponding pretreatments) for 4 hours (without host cells). After treatment, parasites (2.5×10^7^) were collected and extensively washed before LC-MS or GC-MS analysis to measure the relative levels of ^13^C labeled metabolites. To examine the incorporation of glucose derived carbon into fatty acids, parasites were used to infect HFF cells and then cultured in glucose free DMEM supplemented with 8 mM ^13^C_6_-glucose for 48 hours with or without ATc. Subsequently the parasites were forced to egress by needle passage and 2.5×10^7^ purified tachyzoites were subject to GC-MS analysis. Other steps of GC-MS analysis were performed as previously described (2).

For UPLC-MS analysis, parasites suspended in 1 ml of 50% methanol (methanol : ddH_2_O = 1:1) was processed by 5 cycles of 1 min ultra-sonication plus 1 min interval in iced water bath. Then the samples were spun at 16, 000 g for 15 min at 4 °C. Supernatants were collected and evaporated to dryness under nitrogen gas. The residue was then reconstituted in 50 μL of 50% aqueous acetonitrile and used in UPLC-MS analysis. UPLC-MS was performed on a Waters Acquity UPLC system (Waters, Milford, MA, USA) coupled to a Triple Quad™ 5500 tandem mass spectrometer (AB Sciex, Framingham, MA, USA). The samples were separated using a Waters UPLC BEH Amide column (100 mm× 2.1 mm, 1.7 μm) with a linear gradient elution system of mobile-phase. Elution buffers were A (10 mM ammonium acetate and 0.3% ammonium hydroxide in water) and B (10 mM ammonium acetate and 0.3% ammonium hydroxide in acetonitrile/water (90/10, v/v). The gradient elution conditions were 0-1 min, 95% B; 2 min, 70% B; 6 min, 60% B; 6.1 min, 50% B; 7 min, 50%B; 7.1 min, 95%B; 12 min, 95%B. The column temperature was 30 °C and the injection volume was 5 μL. The flow rate was maintained at 300 μL/min. The analytes separated from column were ionized in an electro spray ionization source in negative mode with the following settings: source temperature: 500°C, curtain gas: 25 psi, ion source gas 1: 50 psi, ion source gas 2: 50 psi, collision gas: 8 psi, ion spray voltage: -4500V, entrance potential: -10 V, collision cell exit potential: -10V. The dwelling time was set at 20 ms. Multiple reaction monitoring was used to acquire data in optimized MRM transition (precursor > product), declustering potential (DP), and collision energy (CE). The AB Sciex Analyst software (version 1.5.2) (AB Sciex, MA, USA) was used to control instruments and acquire and analyze data. Analyst (version 1.5.2) (AB Sciex, MA, USA) was used to analyze data. The default parameters and manual inspection were used to ensure the qualitative and quantitative accuracy of each compound, extract and output chromatographic retention time and peak area.

**Determination of parasite burden in peritoneal fluids of mice.**

Freshly egressed tachyzoites of the iPYK1 strain were purified and then 10^4^ parasites were used to infect (two groups, each with three mice) seven-week-old CD1-nude mice (Beijing Vital River Laboratory Animal Technology Co., Ltd, Beijing China) by intraperitoneal injection. Subsequently, the experimental group was administrated with 0.2 mg/ml ATc in drinking water, whereas the control group was given 1.25% ethanol in drinking water. Seven days post infection, peritoneal fluids were collected from infected mice and genomic DNA was extracted from them using the EasyPure Genomic DNA Kit (Transgen Biotech, Beijing, China). Parasite burden in peritoneal fluids was determined by β-tubulin based quantitative PCR, as previously described (2).

**PYK Enzymatic activity assay**

The iPYK1 strain was treated with or without 0.5 μg/ml ATc for two days. Then tachyzoites were purified, harvested and suspended in 100 μl RIPA lysis buffer (Beyotime Biotechnology Co., Ltd, Shanghai, China) to lyse for 15 min by ultrasonication in a water bath sonicator. Supernatants were collected by centrifugation (12,000 g, 5 min) and protein concentration was determined using the BCA protein assay kit (Beyotime Biotechnology Co., Ltd, Shanghai, China). Pyruvate kinase activity in supernatants was monitored by lactate dehydrogenase-coupled spectrophotometric assay, as previously described (5). Briefly, in a 96-well plate format, 0.01 mg parasite lysates were added to each well containing 200 μl reaction buffer (1 mM PEP, 1 mM ADP, 50 mM MgCl_2_, 100 mM KCl, 0.2 mM NADH, 20 units of rabbit muscle lactate dehydrogenase type II (Sigma Aldrich, St. Louis, MO, USA), 50 mM Tris, pH=7.0). Immediately after the initiation of the reaction, the plate was read on a Cytation 5 reader (BioTek instruments, Inc., Winooski, VT, USA) at 340 nm every 30 seconds for 20 minutes. Experiments were carried out in triplicates and PYK enzymatic activity in the lysates was calculated as previously described (5).

**Production of mouse antisera against Toxoplasma PYK2**

The polyclonal antibodies against TgPYK2 were raised in a way similar to what was described before (6). Briefly, the sequence encoding amino acids 293 to 988 of TgPYK2 was amplified from cDNA of the RH strain and cloned into the vector pE-SUMO. Subsequently recombinant His-SUMO-PYK2 (293-988) was purified from *E. coli* BL21(DE3) and used to immunize 6-week-old female ICR mice for antisera production.

**References**

1. **Shen B, Brown K, Long S, Sibley LD.** 2017. Development of CRISPR/Cas9 for Efficient Genome Editing in Toxoplasma gondii. Methods Mol Biol **1498:**79-103.

2. **Xia N, Yang J, Ye S, Zhang L, Zhou Y, Zhao J, David Sibley L, Shen B.** 2018. Functional analysis of Toxoplasma lactate dehydrogenases suggests critical roles of lactate fermentation for parasite growth in vivo. Cell Microbiol **20**.

3. **Shen B, Sibley LD.** 2014. Toxoplasma aldolase is required for metabolism but dispensable for host-cell invasion. Proc Natl Acad Sci U S A **111:**3567-3572.

4. **Nitzsche R, Zagoriy V, Lucius R, Gupta N.** 2016. Metabolic Cooperation of Glucose and Glutamine Is Essential for the Lytic Cycle of Obligate Intracellular Parasite Toxoplasma gondii. J Biol Chem **291:**126-141.

5. **Denton H, Roberts CW, Alexander J, Thong KW, Coombs GH.** 1996. Enzymes of energy metabolism in the bradyzoites and tachyzoites of Toxoplasma gondii. FEMS Microbiol Lett **137(1)**:103-8.

6. **Saito T, Nishi M, Lim MI, Wu B, Maeda T, Hashimoto H, Takeuchi T, Roos DS, Asai T.** 2008. A novel GDP-dependent pyruvate kinase isozyme from Toxoplasma gondii localizes to both the apicoplast and the mitochondrion. J Biol Chem **283:**14041-14052.
