# Supplementary material for: Pyruvate Homeostasis as a Determinant of Parasite Growth and Metabolic Plasticity in Toxoplasma gondii
Source: mBio. 2019 Jun 11;10(3):e00898-19. doi: 10.1128/mBio.00898-19 (PMC6561023; doi:10.1128/mBio.00898-19)
Supplement: TABLE S2 [file mBio.00898-19-st002.docx]

**Table S2-1. Plasmids used in this study**

| Plasmid name | Use | Source |
| --- | --- | --- |
| pSAG1-Cas9- sgUPRT | Template for gene specific CRISPR plasmid construction | Reference 1 |
| pSAG1-Cas9-sgPYK1 | *PYK1* specific CRISPR plasmid for PYK1 knockout | This work |
| pSAG1-Cas9-sgPYK1-cKO | *PYK1* specific CRISPR plasmid for the iPYK1 construction | This work |
| pSAG1-Cas9-sgPYK2 | *PYK2* specific CRISPR plasmid | This work |
| pSAG1-Cas9-sgGT1 | *GT1* specific CRISPR plasmid | This work |
| pTet-off:: PYK1-Ty | To construct the iPYK1 conditional knockdown strain | This work |
| pPYK1::*DHFR* | Homologous template for *PYK1* replacement by *DHFR* | This work |
| pPYK2::*DHFR* | Homologous template for *PYK2* replacement by *DHFR* | This work |
| pPYK2::*CAT* | Homologous template for *PYK2* replacement by *CAT* | This work |
| pGT1::*CAT* | Homologous template for *GT1* replacement by *CAT* | This work |
| pTub::PYK2(cyto)::*CAT* | To construct the iPYK1/PYK2(cyto) strain | This work |
| pTub::PYK2(apico)::*CAT* | To construct the iPYK1/PYK2(apico) strain | This work |
| pLDH1::CAT | Homologous template for *LDH1* replacement by *CAT* | Reference 2 |
| pTub::PYK1::HA::*CAT* | To construct the iPYK1 comp strain | This work |
| p7TetOS1 | Template for SAG1-TetO7 promoter amplification | From the Sibley Lab |
| pUC19 | Template for pUC19 amplification | From the Sibley Lab |

References:

1. **Shen B, Brown KM, Lee TD, Sibley LD.** Efficient gene disruption in diverse strains of Toxoplasma gondii using CRISPR/CAS9. mBio 5, e01114-01114 (2014).
2. **Xia N, Yang J, Ye S, Zhang L, Zhou Y, Zhao J, David Sibley L, Shen B.** Functional analysis of Toxoplasma lactate dehydrogenases suggests critical roles of lactate fermentation for parasite growth in vivo. Cell Microbiol 20 **(**2018).

**Table S2-2. Transgenic parasites used in this study**

| Strain | Description | Source |
| --- | --- | --- |
| TATi | Parental strain | Reference 1 |
| iPYK1 | PYK1 conditional knockdown | This work |
| RH *Δhxgprt* | Parental strain | From the Sibley Lab |
| RH *Δpyk2* | PYK2 deleted in RH *Δhxgprt* | This work |
| iPYK1-*Δpyk2* | PYK2 deleted in iPYK1 | This work |
| iPYK1-*Δgt1* | GT1 deleted in iPYK1 | This work |
| iPYK1/PYK2(cyto) | Truncated PYK2 (AA 358-988) expressed in iPYK1 | This work |
| iPYK1/PYK2(apico) | Full-length PYK2 expressed in iPYK1 | This work |
| iPYK1-*Δldh1* | LDH1 deleted in iPYK1 | This work |
| iPYK1 comp | PYK1-HA expressed in iPYK1 | This work |
| ME49 | Parental strain | From the Sibley Lab |
| ME49 *Δldh1Δldh2* | *LDH1* and *LDH2* deleted in ME49 | Reference 2 |

References:

1. **Meissner M, Schluter D, Soldati D.** Role of Toxoplasma gondii myosin A in powering parasite gliding and host cell invasion. Science 298, 837-840 (2002).

2. **Xia N, Yang J, Ye S, Zhang L, Zhou Y, Zhao J, David Sibley L, Shen B.** Functional analysis of Toxoplasma lactate dehydrogenases suggests critical roles of lactate fermentation for parasite growth in vivo. Cell Microbiol 20 **(**2018).
